# Cost-Effectiveness of Interventions to Promote Physical Activity: A Modelling Study

**DOI:** 10.1371/journal.pmed.1000110

**Published:** 2009-07-14

**Authors:** Linda J. Cobiac, Theo Vos, Jan J. Barendregt

**Affiliations:** Centre for Burden of Disease and Cost-Effectiveness, School of Population Health, The University of Queensland, Herston, Queensland, Australia; University of Cambridge, United Kingdom

## Abstract

**Background:**

Physical inactivity is a key risk factor for chronic disease, but a growing number of people are not achieving the recommended levels of physical activity necessary for good health. Australians are no exception; despite Australia's image as a sporting nation, with success at the elite level, the majority of Australians do not get enough physical activity. There are many options for intervention, from individually tailored advice, such as counselling from a general practitioner, to population-wide approaches, such as mass media campaigns, but the most cost-effective mix of interventions is unknown. In this study we evaluate the cost-effectiveness of interventions to promote physical activity.

**Methods and Findings:**

From evidence of intervention efficacy in the physical activity literature and evaluation of the health sector costs of intervention and disease treatment, we model the cost impacts and health outcomes of six physical activity interventions, over the lifetime of the Australian population. We then determine cost-effectiveness of each intervention against current practice for physical activity intervention in Australia and derive the optimal pathway for implementation. Based on current evidence of intervention effectiveness, the intervention programs that encourage use of pedometers (Dominant) and mass media-based community campaigns (Dominant) are the most cost-effective strategies to implement and are very likely to be cost-saving. The internet-based intervention program (AUS$3,000/DALY), the GP physical activity prescription program (AUS$12,000/DALY), and the program to encourage more active transport (AUS$20,000/DALY), although less likely to be cost-saving, have a high probability of being under a AUS$50,000 per DALY threshold. GP referral to an exercise physiologist (AUS$79,000/DALY) is the least cost-effective option if high time and travel costs for patients in screening and consulting an exercise physiologist are considered.

**Conclusions:**

Intervention to promote physical activity is recommended as a public health measure. Despite substantial variability in the quantity and quality of evidence on intervention effectiveness, and uncertainty about the long-term sustainability of behavioural changes, it is highly likely that as a package, all six interventions could lead to substantial improvement in population health at a cost saving to the health sector.

*Please see later in the article for Editors' Summary*

## Abstract 2

Linda Cobiac and colleagues model the costs and health outcomes associated with interventions to improve physical activity in the population, and identify specific interventions that are likely to be cost-saving.

## Editors' Summary

**Background:**

The human body needs regular physical activity throughout life to stay healthy. Physical activity—any bodily movement produced by skeletal muscles that uses energy—helps to maintain a healthy body weight and to prevent or delay heart disease, stroke, type 2 diabetes, colon cancer, and breast cancer. In addition, physically active people feel better and live longer than physically inactive people. For an adult, 30 minutes of moderate physical activity—walking briskly, gardening, swimming, or cycling—at least five times a week is sufficient to promote and maintain health. But at least 60% of the world's population does not do even this modest amount of physical activity. The daily lives of people in both developed and developing countries are becoming increasingly sedentary. People are sitting at desks all day instead of doing manual labor; they are driving to work in cars instead of walking or cycling; and they are participating less in physical activities during their leisure time.

**Why Was This Study Done?:**

In many countries, the chronic diseases that are associated with physical inactivity are now a major public-health problem; globally, physical inactivity causes 1.9 million deaths per year. Clearly, something has to be done about this situation. Luckily, there is no shortage of interventions designed to promote physical activity, ranging from individual counseling from general practitioners to mass-media campaigns. But which intervention or package of interventions will produce the optimal population health benefits relative to cost? Although some studies have examined the cost-effectiveness of individual interventions, different settings for analysis and use of different methods and assumptions make it difficult to compare results and identify which intervention approaches should be give priority by policy makers. Furthermore, little is known about the cost-effectiveness of packages of interventions. In this study, the researchers investigate the cost-effectiveness in Australia (where physical inactivity contributes to 10% of deaths) of a package of interventions designed to promote physical activity in adults using a standardized approach (ACE-Prevention) to the assessment of the cost-effectiveness of health-care interventions.

**What Did the Researchers Do and Find?:**

The researchers selected six interventions for their study: general practitioner “prescription” of physical activity; general practitioner referral to an exercise physiologist; a mass-media campaign to promote physical activity; the TravelSmart car use reduction program; a campaign to encourage the use of pedometers to increase physical activity; and an internet-based program. Using published data on the effects of physical activity on the amount of illness and death caused by breast and colon cancer, heart disease, stroke, and type 2 diabetes and on the effectiveness of each intervention, the researchers calculated the health outcomes of each intervention in disability-adjusted life years (DALY; a year of healthy life lost because of premature death or disability) averted over the lifetime of the Australian population. They also calculated the costs associated with each intervention offset by the costs associated with the five conditions listed above. These analyses showed that the pedometer program and the mass-media campaign were likely to be the most cost-effective interventions. These interventions were also most likely to be cost-saving. Referral to an exercise physiologist was the least cost-effective intervention. The other three interventions, though unlikely to be cost-saving, were likely to be cost-effective. Finally, a package of all six interventions would be cost-effective and would avert 61,000 DALYs, a third of what could be achieved if every Australian did 30 minutes of physical activity five times a week.

**What Do These Findings Mean?:**

As in all modeling studies, these findings depend on the quality of the data and on the assumptions included by the researchers in their calculations. Unfortunately, there was substantial variability in the quantity and quality of evidence on the effectiveness of each intervention and uncertainty about the long-term effects of each intervention. Nevertheless, the findings presented in this study suggest that the assessment of the cost-effectiveness of a combination of interventions designed to promote physical activity might provide policy makers with some guidance about the best way to reduce the burden of disease caused by physical inactivity. More specifically, for Australia, these findings suggest that the package of the six interventions considered here is likely to provide a cost-effective way to substantially improve the health of the nation.

**Additional Information:**

Please access these Web sites via the online version of this summary at http://dx.doi.org/10.1371/journal.pmed.1000110.

- The World Health Organization provides information about physical activity and health (in several languages); it also provides an explanation of DALYs
- The US Centers for Disease Control and Prevention provides information on physical activity for different age groups and for health professionals
- The UK National Health Service information source Choices also explains the benefits of regular physical activity
- MedlinePlus has links to other resources about exercise and physical fitness (in English and Spanish)
- The University of Queensland Web site has more information on ACE-Prevention (Assessing Cost-Effectiveness Prevention)

## Introduction

Physical activity occurs during work, transport, domestic, and leisure-time activities. Too little physical activity increases the risks of ischaemic heart disease, stroke, colon cancer, breast cancer, and type 2 diabetes [1], as well as obesity [2] and falls in later life [3]. The World Health Organization recommends at least 30 minutes of regular, moderate-intensity physical activity on most days to reduce the risk of disease and injury [4].

Lack of physical activity is a problem in many developed countries, and a growing concern for developing countries adopting a progressively “Westernised” lifestyle [5]. Australia is no exception, with only 44% of men and 36% of women achieving sufficient physical activity for health [6]. This inactivity contributes 7% of Australia's disease burden and 10% of all deaths, mostly due to cardiovascular disease and diabetes [7]. It also places a substantial burden on the Australian economy through the costs of treatment for physical activity–related disease and injury, lost productivity, and diminished quality of life [8].

Interventions to promote physical activity (referred to herein as “physical activity interventions”) typically involve teaching individuals the skills to change physical activity behaviour, providing the population with knowledge about physical activity goals or opportunities to be active, or creating a more physically active environment [9]. Currently, in Australia, general practitioners (GPs) are relied upon to deliver physical activity interventions (when time permits). In addition, governments provide some encouragement to change physical activity behaviour through local mass media and transport campaigns, but investment is minimal. It is likely that a combination of intervention approaches will be needed to achieve a meaningful change in population participation in physical activity [10]–[12].

Efficient allocation of health resources to different intervention programs hinges on identifying those interventions (or combinations of interventions) that achieve maximum population health benefits relative to cost. However, there is a need for more cost-effectiveness analyses using standardised methods to enable comparison of different types of physical activity interventions [13]. Although there have been a number of cost-effectiveness analyses of individual interventions [14]–[20], including two studies reporting cost-effectiveness of GP prescription in the Australian context [17],[19], there has been only one cost-effectiveness analysis, by the National Institute of Clinical Excellence (NICE) in the United Kingdom [21], that used a standardised approach to compare multiple interventions. All ten interventions, which involved brief advice by a health professional or referral to an exercise physiologist, were found to be dominant (i.e., cost-saving) when compared with usual care. However, the NICE study focused only on intervention alternatives rather than evaluating a range of complementary interventions (including broader public health approaches incorporating media, active transport, etc.) and the potential benefits of combining interventions as a package.

In Australia, a standardised approach to assessing cost-effectiveness (ACE) has been developed for evaluating interventions in the Australian health care context [22]. These methods are being used to evaluate 150 interventions, focusing on prevention of noncommunicable disease, including six interventions to promote physical activity. In this paper, we present the ACE results for the physical activity interventions, which range from individualised counselling interventions to broad population health approaches. The cost-effectiveness of each intervention is compared with current practice for physical activity intervention in the Australian population, and from this we derive an optimal intervention pathway for improving population health.

## Methods

### Interventions

We reviewed the physical activity and transport intervention literature to identify a range of interventions targeting the adult population, which would be suitable for implementation in Australia, and had evidence of efficacy/effectiveness to support the analyses. Where there were multiple studies of the same type of intervention, studies were combined in a meta-analysis, using a random effects approach where there was heterogeneity between trial results. However, where multiple studies were too heterogeneous to enable a precise definition of the intervention and comparator (i.e., who did what, to whom, when, and where) and accurate measurement of resources used, a single study was chosen for economic evaluation. This selection was based on the strength of evidence of effectiveness and generalisability of the setting and population to the Australian context (i.e., giving consideration to both the internal and external validity of the intervention evidence). A full description of the review and criteria for selection of interventions is provided in Text S1. From the review process, we selected six intervention programs for cost-effectiveness analysis:

#### GP prescription

Patients are screened opportunistically when visiting their general practice; inactive patients receive a physical activity prescription from the GP and follow-up phone call(s) from an exercise physiologist.

#### GP referral to exercise physiologist

Screening questionnaires are mailed to all patients on the GP patient list; inactive patients are invited to attend a series of counselling sessions with an exercise physiologist at their local general practice.

#### Mass media-based campaign

A six-week campaign combines physical activity promotion via mass media (television, radio, newspaper, etc.), distribution of promotional materials, and community events and activities.

#### TravelSmart

An active transport program targets households with tailored information (e.g., maps of local walking paths, bus timetables) and merchandise (e.g., water bottles, key rings) as an incentive and/or reward for reducing use of cars for transport.

#### Pedometers

A community program encourages use of pedometers as a motivational tool to increase physical activity (e.g., to 10,000 steps per day).

#### Internet

Participants are recruited via mass media to access physical activity information and advice across the internet via a Web site and/or email.

Table 1 gives a summary of the intervention effects, costs, and target groups. The effect of each intervention on physical activity in the Australian population is derived from the characteristics of the intervention target group (e.g., age, sex, and baseline physical activity participation) and intervention changes in intensity, duration, and/or frequency of physical activity. The cost of implementing each intervention is derived from an Australian health sector perspective. This includes costs to both government and patients, including time and travel costs, but excluding patient time costs associated with changes in physical activity. Intervention start-up costs (e.g., costs of research and development of intervention materials for GP prescription) are excluded so that all interventions are evaluated and compared as if operating under steady-state conditions (i.e., fully implemented and operating in accordance with effectiveness potential). Further details of each intervention are provided in Text S1.

**Table 1:** The target groups, physical activity effects, and costs associated with implementing the physical activity interventions in Australia for one year (2003 baseline year).

Intervention	Target Group^a^	Effect in Target Group	Cost (AUS$million)^b^	Sources
GP prescription	25% of sedentary and 10% of insufficiently active population aged 40–79 y	160 MET-min/wk	$250 intervention; $32 time and travel	Target group derived from RCT recruitment rates [36] and Australian GP statistics. Effect derived from Δkcal/kg/wk observed in the RCT. Costs adapted from New Zealand study [24].
GP referral to exercise physiologist	8% of sedentary and 3% of insufficiently active population aged 60+ y	238 MET-min/wk	$190 intervention; $160 time and travel	Target group derived from RCT recruitment rates [43] and Australian GP statistics. Effect derived from Δsessions/wk and Δmin/session observed in RCT [44]. Total costs estimated from resource use (e.g., screening questionnaire printing/delivery, exercise physiologist salary, etc.)
Mass media-based campaign	100% of population aged 25–60 yrs	148 MET-min/wk	$13 intervention; $0 time and travel	Target population based on population in quasi-experimental study of Australian campaign [45]. Effect derived from Δh/wk observed in the Australian campaign. Costs estimated from similar Australian campaign [46].
Internet	2% of population (internet users) aged 15+ y	129 MET-min/wk	$21 intervention; $0 time and travel	Target group derived from participation and attrition rates in 3 RCTs [47]–[49] and Australian internet access statistics. Effect derived from meta-analysis of ΔMET-min/wk in the 3 RCTs. Costs estimated from costs for operating similar health Web site in Victoria.
Pedometers	13% of population aged 15+ y	574 MET-min/wk	$53 intervention; $0 time & travel	Target group derived from participation rates observed in the Rockhampton 10,000 steps program [50]. Effect derived from Δsteps/d from meta-analysis of 8 RCTs [51]. Costs derived from weighted average of resource use in the 8 RCTs and costs of Rockhampton program [35].
TravelSmart	57% of population (urban) aged 15+ y	57 MET-min/wk	$412 intervention; $0 time and travel	Target population derived from household contact rates in 21 TravelSmart studies. Effect derived as weighted average of Δtrips/wk (walking/cycling) observed in the TravelSmart studies. Costs derived from costs of TravelSmart intervention delivery in Western Australia.

a Activity definitions for intervention analysis: sedentary (<100 MET-min/wk), insufficiently active (<750 MET-min/wk), and sufficiently active (≥750 MET-min/wk ≈30 min of activity on 5 d of the week at a “moderate” intensity of 5 METs, i.e., 5× resting metabolic rate).

b All costs are adjusted to real prices in the 2003 reference year using the relevant Health Price Index from the Australian Institute of Health and Welfare [52], or relevant Consumer Price Index from the Australian Bureau of Statistics [53] where the costs would occur outside of the health sector.

### Modelling Health Outcomes and Costs

Cost-effectiveness analysis is based on intervention in the first year, with all health outcomes and costs measured over the lifetime of the Australian population in a baseline year of 2003. All future health outcomes and costs are discounted at 3% per annum.

The health outcomes of each intervention are evaluated in disability-adjusted life years (DALYs), the measure favoured by the World Health Organization [23], and the alternative to the quality-adjusted life year (QALY) measure used in some cost-effectiveness analyses of physical activity interventions [21],[24]. The critical difference between the DALY and QALY measures is in the measurement of utility weights for the QALY and disability weights for the DALY. Utility weights are typically elicited from general population samples or groups of patients, and do not always match the specific disease and physical activity states used in modelling cost-effectiveness of interventions, a limitation acknowledged by Roux et al. [25] in their recent evaluation of physical activity interventions using QALY measures. Utility weights also lack consistency across many different diseases. Although techniques for eliciting disability weights for each disease are controversial [26], the use of a standard set of weights across all diseases has advantages in large projects, where cost-effectiveness decision-making encompasses many disease and risk factor interventions (e.g., Australia's ACE–Prevention project) and sometimes many regions of the world (e.g., World Health Organization's WHO-CHOICE [27]).

DALYs are calculated using a multi-state, multiple cohort life-table approach to determine changes in mortality and morbidity for five physical activity-related diseases: ischaemic heart disease, ischaemic stroke, type 2 diabetes, breast cancer, and colon cancer. The effects of physical activity on other risk factors (e.g., obesity and falls), which require more complex modelling, and the effects of physical activity on *prevention* of depression, which is still a subject of debate [28], are being evaluated in separate ACE modelling analyses and results are not presented here.

The cost of each intervention is offset by the cost per incident case of breast cancer and colon cancer averted and the cost per prevalent case of ischaemic heart disease, ischaemic stroke, and type 2 diabetes averted. Health care costs for all other diseases in added years of life are excluded from basic results, but their influence on cost-effectiveness is explored in additional analyses, as recommended by the Panel on Cost-Effectiveness in Health and Medicine in the United States [29].

The intervention effects on disease risk have previously been modelled from observed changes in prevalence of physical activity across two or more categories, with relative risks derived for each intervention from health outcome studies using comparable physical activity categories (e.g., [21]). The two key drawbacks to this approach are that it limits evaluation to intervention studies that measure categorical outcomes (e.g., active/inactive) and precludes analysis of intervention combinations to calculate an optimal intervention pathway, because categories are rarely defined the same way in different intervention studies. We instead estimate the change in relative risk of each physical activity–related disease from a change in energy expenditure, which we derive from estimates of activity intensity, duration, and/or frequency for each intervention (Text S1).

The relationships between relative risk and energy expenditure are derived for each physical activity–related disease from meta-analyses carried out for the World Health Organization's *Comparative Quantification of Health Risks* [1]. Relative risks are assumed to decrease linearly with increasing energy expenditure, up to the level of physical activity at which there is no excess risk (≈30 min of activity on 5 d of the week at a moderate intensity of 5 METs, i.e., 5× the resting metabolic rate). Full details of these modelling methods and assumptions, and all data sources, are included in Text S2.

### Intervention Cost-Effectiveness

A cost-effectiveness ratio (CER) is evaluated for each intervention in comparison with current practice, which approximates a “do nothing” scenario. Our review of intervention implementation in Australia found that while some interventions were in place in 2003, all were operating at less than 5% of the estimated full capacity (Table 2).

**Table 2:** Current practice for the six physical activity interventions in 2003.

Intervention	Current Capacity (Percentage of Full Capacity)	Assumptions
GP prescription	1.2%	1.5 per 100 encounters involve exercise counselling/advice [54]; 16.3% of GPs provide written information [55]; adjusted for number of GP encounters at age 40–79 y [54].
GP referral to exercise physiologist	3.6%	1.5 per 100 encounters involve exercise counselling/advice [54]; 13.2% of GPs provide referral to qualified exercise personnel [55]; adjusted for number of GP encounters at age 60+ y [54].
Mass media-based campaign	1.0%	Population exposed to mass media-based campaign in NSW in 1998 [45]; assuming 50% decay in intervention effect per year.
TravelSmart	1.2%	Population in suburbs that received intervention up to and including 2003 – Melville, Perth (pilot), Perth, Grange, Marangaroo, Cambridge, Subiaco, Fremantle, Armadale, Vincent and Alamein.
Pedometers	0.3%	Australian population, aged 15+ years in 2003 [56], exposed to Rockhampton 10,000 steps intervention [57].
Internet	0%	No evidence located for internet-based interventions in practice in Australia in 2003.

Ninety-five percent uncertainty intervals are determined for all outcome measures by Monte Carlo simulation (2,000 iterations), using the Excel add-in tool @RISK (Palisade, Version 4.5). Uncertainty distributions around input parameters are described in Text S3.

The results of the Monte Carlo analysis are used to determine probability of intervention cost-effectiveness against a range of threshold values. In this paper, results are reported against a cost-effectiveness threshold of AUS$50,000 per DALY [30],[31].

### Intervention Pathway Analysis

The optimal pathway for implementation of interventions is developed using a generalised cost-effectiveness approach [32]. We first derive the disease incidence rates that would have occurred in 2003 if none of the interventions under evaluation (Table 2) were in place. This scenario is referred to as the “partial null.” The cost-effectiveness of each intervention is then evaluated in comparison with the partial null to determine the order of interventions in the pathway, from most cost-effective to least cost-effective. Finally, the cost-effectiveness of each intervention combination in the pathway is evaluated in comparison with the partial null. From this we derive an *incremental* cost-effectiveness ratio (ICER) for each intervention, which reflects the cost-effectiveness of adding the intervention to the pathway.

### Sensitivity Analysis

The sustainability of intervention health effects over time is an important parameter in the cost-effectiveness analysis, but there are currently too few studies with long-term participant follow-up (e.g., greater than two years) to quantify the sustainability of the physical activity effect associated with each of the interventions. In our base case analysis we assume that the intervention effects on physical activity are sustained for the first year, but decay exponentially at a rate of 50% per annum thereafter; therefore, there will be virtually no intervention effect after five years. Sensitivity of the intervention pathway to this assumption is evaluated by varying decay rates between 0% (lifelong behaviour change) and 100% (behaviour change reversed after the first year).

## Results

### Intervention Cost-Effectiveness

There is large variability in the health gain that can be achieved with different methods of intervention. The number of DALYs averted ranges from 740 (95% uncertainty interval [UI] 110–1,900) for internet-based intervention to 23,000 (95%UI 7,600–40,000) for a mass media campaign (Table 3). Intervention costs also vary substantially, ranging from AUS$13 million (95%UI AUS$11 million to 16 million) for the mass media campaign to AUS$410 million (95%UI AUS$210 million to 570 million) for the TravelSmart individualised marketing program.

**Table 3:** Cost-effectiveness of physical activity interventions when compared with current practice.

Intervention	DALYs Averted	Cost Offsets (AUS$million)	Intervention Cost (AUS$million)	Net Cost (AUS$million)	Median ICER (AUS$/DALY)
Pedometers	20,000 (9,100 to 33,000)	−$480 (−$820 to −$200)	$54 ($4.0 to $170)	−$420 (−$780 to −$120)	Dominant (Dominant to Dominant)
Mass media	23,000 (7,600 to 40,000)	−$440 (−$820 to −$140)	$13 ($11 to $16)	−$430 (−$800 to −$130)	Dominant (Dominant to Dominant)
TravelSmart	9,300 (−1,400 to 22,000)	−$220 (−$550 to $31)	$410 ($210 to $570)	$190 (−$120 to $490)	$18,000 (Dominant to $330,000)
GP prescription	7,100 (1,000 to 13,000)	−$170 (−$340 to −$26)	$250 ($190 to $310)	$81 (−$80 to $240)	$11,000 (Dominant to $140,000)
GP referral	1,900 (1,000 to 3,000)	−$54 (−$94 to −$27)	$190 ($150 to $240)	$140 ($94 to $180)	$75,000 ($37,000 to $150,000)
Internet	740 (110 to 1,900)	−$17 (−$45 to −$2.4)	$21 ($2.0 to $64)	$3.0 (−$33 to $51)	$2,000 (Dominant to $210,000)

**Figure 1:**
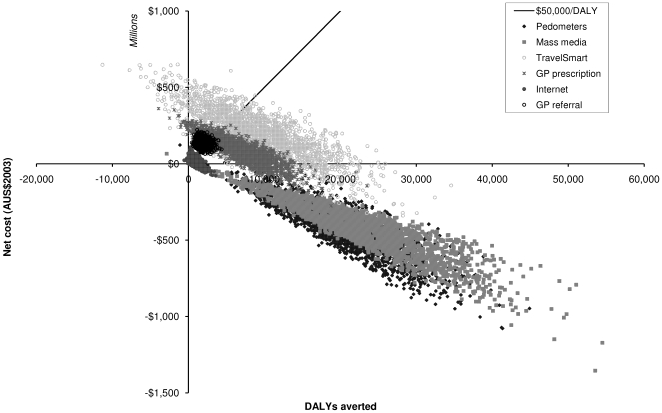
Cost-effectiveness of the physical activity interventions when compared with current practice.

The interventions predominantly fall in the northeast and southeast quadrants of the cost-effectiveness plane (Figure 1), indicating a high probability of improvements in population health with increased expenditure on physical activity intervention or, in some cases, with a net cost-saving due to physical activity intervention.

Two interventions stand out as being most effective and most cost-effective—the mass media campaign and the pedometer program. Both of these interventions are dominant and have a 100% probability of being cost-saving (Table 4).

**Table 4:** Acceptability of physical activity interventions when compared with current practice.

Intervention	Probability of Being Cost-Saving	Probability of Being<AUS$50,000/DALY
Pedometers	100%	100%
Mass media	100%	100%
TravelSmart	10%	74%
GP prescription	15%	89%
GP referral	0%	13%
Internet	47%	84%

Only the GP referral intervention has a low probability of being under the AUS$50,000 per DALY cost-effectiveness threshold when all costs are considered. GP referral has a substantial time and travel cost component for patients in visiting an exercise physiologist. If these costs are excluded from the analysis, the intervention is dominant, with a 100% probability of being under the threshold and a 98% probability of being cost-saving.

### Intervention Pathway

**Figure 2:**
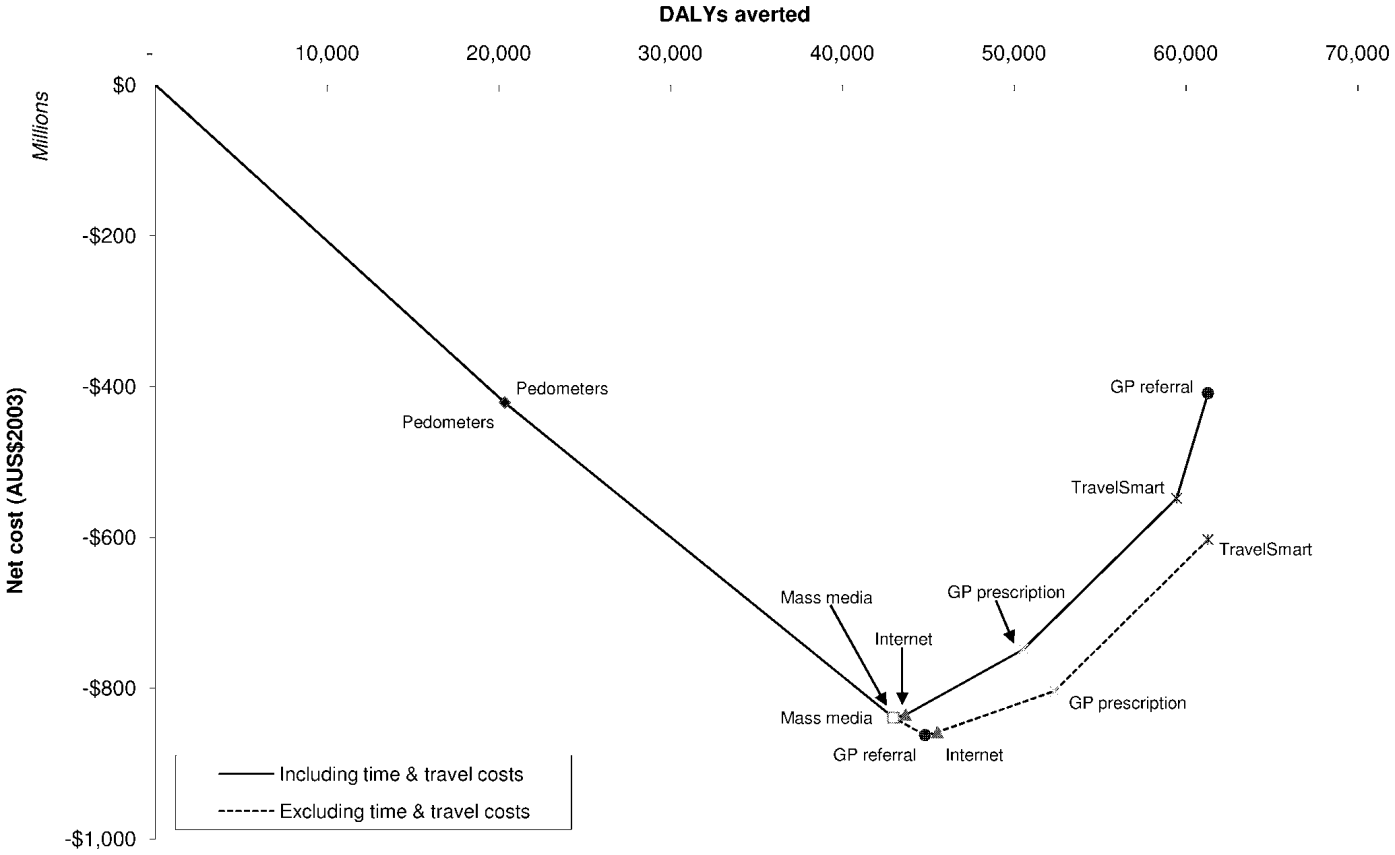
The physical activity intervention pathway.

When all six interventions are combined in a package, the package would avert 61,000 DALYs (95%UI 39,000–87,000 DALYs). This is 34% of what could theoretically be achieved if all Australians (except the most disabled) achieved the sufficient level of activity recommended by the World Health Organization [4] and Australian Physical Activity Guidelines [33]. The health gain from the six interventions would be achieved at a total cost of AUS$940 million (95%UI AUS$720 million to 1,100 million), which includes AUS$90 million in time and travel costs. However, the costs of intervention would be more than offset by an estimated reduction of AUS$1,400 million (95%UI AUS$790 million to 2,100 million) in the costs of treating physical activity-related diseases. The cost-effectiveness of the pathway is reflected by its location in the southeast quadrant of the cost-effectiveness plane (Figure 2).

In order of cost-effectiveness, a pedometer intervention program should be implemented first, followed by a mass media campaign, an internet-based program, GP prescription, the TravelSmart program, and, finally, GP referral to an exercise physiologist. In this pathway, only GP referral has a low probability of being under the AUS$50,000 per DALY threshold for cost-effectiveness (Table 5). Exclusion of time and travel costs, which greatly affect the cost of GP referral, shifts the intervention from last place up to third position in the order, with the sequence of all other interventions remaining the same. Including health care costs in added years of life, for all diseases and injuries other than those explicitly modelled, leads to less favourable cost-effectiveness results, but the effect is relatively minor. This is because the average increase in life expectancy in the whole population is relatively small (up to 6 d, with a 50% decay in intervention effects), and the costs are incurred at the end of the lifespan, with future costs discounted back to the baseline year. Overall, the change in cost-effectiveness is not sufficient to alter decision-making about implementation of the physical activity interventions.

**Table 5:** Incremental cost-effectiveness of interventions in the physical activity pathway.

Intervention	Excluding Costs of Unrelated Health Care^a^ in Added Years of Life			Including Costs of Unrelated health Care^a^ in Added Years of Life		
	Median ICER (AUS$/DALY)	Probability of Being Cost-Saving	Probability of Being<AUS$50,000/DALY	Median ICER (AUS$/DALY)	Probability of Being Cost-Saving	Probability of Being<AUS$50,000/DALY
Pedometers	Dominant (Dominant–Dominant)	100%	100%	Dominant (Dominant–Dominant)	99%	100%
Mass media	Dominant (Dominant–Dominant)	100%	100%	Dominant (Dominant–Dominant)	100%	100%
Internet	$3,000 (Dominant–$210,000)	46%	83%	$10,000 (Dominant–$220,000)	34%	80%
GP prescription	$12,000 (Dominant–$150,000)	13%	88%	$20,000 (Dominant–$150,000)	2%	85%
TravelSmart	$20,000 (Dominant–$350,000)	8%	73%	$27,000 (Dominant–$360,000)	2%	69%
GP referral	$79,000 ($39,000–$150,000)	0%	10%	$89,000 ($49,000–$160,000)	0%	3%

Each incremental cost-effectiveness ratio represents the cost-effectiveness of adding the intervention to the package (i.e., it does not represent progressive cost-effectiveness of the intervention package compared with the partial null “no intervention” scenario).

a The “costs of unrelated health care” includes all costs for disease and injury other than for the physical activity-related diseases explicitly modelled.

### Pathway Sensitivity

**Figure 3:**
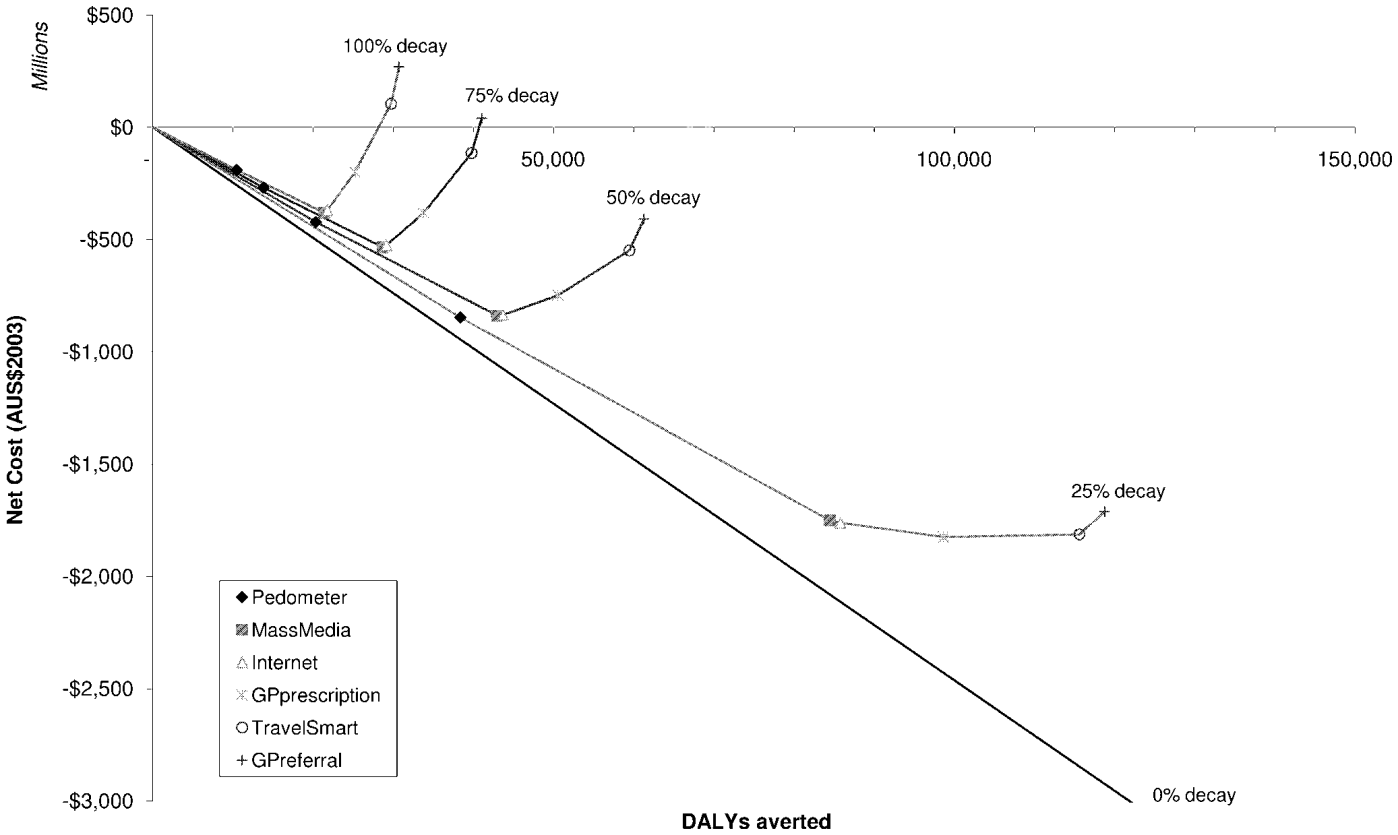
Sensitivity of the intervention pathway to the rate of decay in intervention health effects.

The more quickly the intervention effects on physical activity behaviour are assumed to decline over time, the less cost-effective the intervention package becomes (Figure 3). At higher levels of decay, the total package is no longer cost-saving, although it is still under AUS$50,000 per DALY.

The first four interventions in the pathway—pedometers, mass media, internet-based intervention and GP prescription—are under the AUS$50,000 per DALY threshold for cost-effectiveness under all decay scenarios (Table 6). The key differences between the sensitivity scenarios and the base case results are that GP referral is cost-effective when decay is slower than the 50% assumed in the primary analysis, and that the TravelSmart program is no longer cost-effective at maximum (i.e., 100%) decay.

**Table 6:** Sensitivity of the median ICERs to the rate of decay in intervention health effects.

Intervention	Median ICER (AUS$/DALY)				
	0% Decay	25% Decay	50% Decay^a^	75% Decay	100% Decay
Pedometers	Dominant	Dominant	Dominant	Dominant	Dominant
Mass media	Dominant	Dominant	Dominant	Dominant	Dominant
Internet	Dominant	Dominant	$3,000	$15,000	$28,000
GP prescription	Dominant	Dominant	$12,000	$30,000	$48,000
TravelSmart	Dominant	Dominant	$20,000	$41,000	$63,000
GP referral	Dominant	$34,000	$79,000	$120,000	$170,000

a Base-case scenario.

## Discussion

Intervention to encourage an increase in physical activity participation is highly recommended in Australia. Potential reductions in costs of treating ischaemic heart disease, stroke, diabetes, breast cancer, and colon cancer mean that there is a high probability of cost-savings from a health sector perspective. Taken as a package of interventions, all six physical activity interventions could lead to a substantial improvement in population health at under AUS$50,000 per DALY.

Cost-effectiveness of the package is not highly sensitive to the sustainability of behavioural changes (total package is under AUS$50,000 per DALY at maximum rates of decay). However, it is likely that some interventions will lead to a more sustained effect than others, and this could affect the order of implementation in the pathway. It is also possible that there will be synergistic effects with implementation of multiple interventions, which could improve the sustainability of intervention effects on physical activity over time, thus increasing cost-effectiveness of the intervention package. However, this may well be countered by a decrease in effectiveness of each additional intervention, due to the increasing proportion of the population less willing or able to change their physical activity behaviour.

When modelled from the selected studies of intervention effectiveness, intervention programs that encourage use of pedometers and mass media-based community campaigns are the most cost-effective strategies to implement and are very likely to be cost-saving. We found that these interventions have the potential to deliver large health benefits to the population, despite the seemingly small or nonsignificant effects on physical activity *behaviour* when measured at a population level [34],[35].

Overall, our intervention cost-effectiveness ratios, which ranged from dominant up to AUS$75,000 per DALY, were not as favourable as the entirely dominant results reported by NICE for ten GP prescription and referral interventions in the United Kingdom [21]. This may be because the NICE analysis did not include patient costs of time and travel, and assumed a slower decay in physical activity behaviour change (50% of participants assumed to maintain change in behaviour long enough to experience health benefits e.g. 20 y for a 25-y-old), but there were also other differences in modelling methods and assumptions (e.g., discount rates) that may have influenced the more favourable NICE results.

Conversely, our cost-effectiveness ratios were mostly *more* favourable than the cost-effectiveness ratios recently reported for seven physical activity intervention programs in the US [25]. Costs per QALY ranged from US$14,000 (2003AUS$19,000) to US$69,000 (2003AUS$91,000) for the physical activity promotion interventions, which included two community-wide campaigns, two social support walking programs, two individually adapted behaviour change programs, and one program to enhance access to a more active environment (e.g., new bicycle paths, fitness centre, etc.). Some additional costs were included in the US analysis (e.g., patient out-of-pocket expenses for physical activity clothing and equipment) contributing to relatively high intervention costs per person, which may have led to the less favourable US results, but there were also many other differences in analysis methods and assumptions, such as a shorter time horizon (40 y), additional medical inflation on disease costs (8% per annum), and more sustained effects on behaviour (33% to 50% decay in the second year, with maintenance of effect thereafter), which complicate interpretation of the contrasting results.

However, the NICE results, the US study, and our own analyses together provide good evidence that physical activity intervention can be cost-effective in the UK, US, and Australia. Over 20 different intervention programs have now been evaluated in these countries, with only four programs exceeding a cost-effectiveness threshold of AUS$50,000.

To our knowledge, no other studies have evaluated cost-effectiveness of this package of interventions. However, two studies have previously evaluated GP prescription intervention based on the randomised controlled trial of New Zealand's Green Prescription program [36], reporting costs per QALY of NZ$2,100 (2003AUS$2,000) [19] and dominant [21]. Although not directly comparable to each other or to our cost per DALY of AUS$11,000 due to different analysis methods and assumptions (e.g., discount rates), the growing number of analyses reporting cost-effectiveness under varying methods and assumptions strengthens the argument for this particular physical activity intervention as a cost-effective measure for improving public health. However, the results for the Green Prescription program do not necessarily reflect the cost-effectiveness of all physical activity prescription programs (many of which have not shown a significant effect on physical activity behaviour [37]–[40]).

A number of limitations must be taken into account when evaluating the results of this research. For example, because of inconsistent physical activity outcome measures and a limited number of randomised controlled trials, for some interventions we selected single intervention studies for cost-effectiveness analysis rather than combining multiple trials in a meta-analysis. Therefore, while the results reflect the cost-effectiveness of the interventions that were evaluated, and based on the best available evidence, they should not be generalised to all interventions of a similar type.

In addition, although interventions were evaluated as if implemented for one year, in some cases it was necessary to include studies of less than one-year duration. While it would be preferable to include only those studies with follow-up data at one year, this would exclude a number of interventions from cost-effectiveness analysis (e.g., active transport interventions, community mass media and pedometer programs, etc.), and potentially bias cost-effectiveness analyses toward the more targeted interventions (e.g., general practice interventions) for which longer-term studies are more readily available. We have included shorter-duration studies in the interests of modelling a wide range of interventions, but acknowledge that these interventions may not prove to be as cost-effective if subsequent intervention studies find a significant drop in effectiveness at one year.

Furthermore, the level of evidence underlying the measures of intervention effect is relatively weak. For example, evaluation of the mass media campaign effect on population health was based only on the results of a single quasi-experimental study, and evaluation of the pedometer program effect on population health was based on a meta-analysis that included only 277 participants in total. In addition, it is likely that those who volunteered to participate in the pedometer trials were more active or more motivated to change their activity behaviour than the general population, leading to a more favourable estimate of cost-effectiveness than might actually occur with rollout of a pedometer program across Australia. Further randomised controlled trials, using consistent measures of physical activity behaviour, would improve our confidence in both the relative position of interventions in the pathway and the overall magnitude of the health gain that could be achieved.

There are a number of other unknowns in modelling physical activity that may have influenced our cost-effectiveness results. Due to the reliance on (mainly) observational studies in the meta-analyses of relative risks of disease by Bull et al. [1], it is possible that the risk would not be fully reversible for those increasing their physical activity in response to an intervention. It is also plausible that there is a time lag between change in physical activity behaviour and change in risk, which may be relatively short for cardiovascular diseases [41], but longer for cancers [42]. These factors could lead to an overestimate of cost-effectiveness ratios. We do, however, incorporate an attenuation of cancer risk by age (see Table I in Text S2), which Bull et al. [1] based on ischaemic heart disease data, that might be an overestimate of attenuation and may, therefore, offset risk reversibility and lag effects.

Nevertheless, the research illustrates how combining physical activity interventions in a cost-effectiveness expansion pathway can provide guidance to policymakers in identifying the most cost-effective approaches to decreasing the burden of disease due to physical inactivity, based on the best available evidence. For Australia, based on current evidence, it is likely that the package of interventions would not only be cost-effective but very likely cost-saving to the health sector, leading to substantial improvements in health for the Australian population.

## Supporting Information

Text S1 Physical activity interventions.
(0.18 MB DOC)

Text S2 Cost-effectiveness modelling methods.
(0.16 MB DOC)

Text S3 Input parameters and uncertainty.
(0.15 MB DOC)

## Abbreviations

ACE: assessing cost-effectiveness
CER: cost-effectiveness ratio
DALY: disability-adjusted life year
GP: general practitioner
ICER: incremental cost-effectiveness ratio
QALY: quality-adjusted life year
RCT: randomised controlled trial
UI: uncertainty interval
