# Supplementary material for: Cost-Effectiveness of Interventions to Promote Physical Activity: A Modelling Study
Source: PLoS Med. 2009 Jul 14;6(7):e1000110. doi: 10.1371/journal.pmed.1000110 (PMC2700960; doi:10.1371/journal.pmed.1000110)
Supplement: Text S1 — Physical activity interventions. (0.18 MB DOC) [file pmed.1000110.s001.doc]

Physical activity interventions

In choosing interventions for analysis, we have aimed to include a broad array of intervention approaches that could be implemented in Australia. Intervention studies, both published and unpublished, were identified by the first author through a search of PUBMED, EMBASE, The Cochrane Library, and Web of Knowledge (Web of Science and ISI) databases, the grey literature, and bibliographies of relevant studies and reviews, and through information supplied by relevant experts.

We evaluated the strength of evidence of each study using an extended six-level version of the standard evidence classification system (Table I). Ambiguities in the evaluation of evidence were discussed by the first two authors, but a Technical Advisory Panel, consisting of three Australian experts in physical activity research, was also formed to provide additional advice where uncertainties could not be resolved by the authors. Studies were excluded from the evaluation if:

- the intervention did not involve primary prevention in the adult population;
- the study did not have a ‘usual care’ or ‘current practice’ comparator for the cost-effectiveness analysis;
- the evidence of effectiveness was inconclusive or did not exist (Level 5-6 in Table I); or
- the evidence of effectiveness was weak or limited (Level 3-4 in Table I) AND the evidence of effectiveness could not be generalised to another setting or population.

Where there were multiple studies of the same type of intervention, studies were combined in a meta-analysis. However, where multiple studies were too heterogeneous to enable a precise definition of the intervention and comparator (i.e. who did what, to whom, when, and where) and accurate measurement of resources used, a single study was chosen for economic evaluation. This selection was based on the strength of evidence of effectiveness and generalisability of the setting and population to the Australian context (i.e. giving regard to both the internal and external validity of the intervention evidence).

A brief description is provided for each intervention, with an explanation of assumptions used to model population-level physical activity effects, the size of the target group and total costs associated with implementation in the Australian population.

Combining interventions in a cost-effectiveness pathway requires a comparable approach to defining intervention effects on physical activity. Changes in physical activity are derived for all interventions in MET-minutes per week[[1]](#footnote-2) from observed changes in intensity, duration and/or frequency of activity. Unless otherwise specified, activity intensities of 3.5 METS of walking, 5.0 METS for moderate activity and 7.5 METS for vigorous activity are assumed, which is consistent with definitions of intensity in the National Health Survey data that we use to define physical activity participation in Australia [1].

Table I: Classification of the strength of evidence [2]. This system is based on the traditional classification of evidence according to epidemiological study design, with modifications to incorporate more indirect and parallel forms of evidence (e.g. information that strongly and logically suggests than an effect exists, or evidence of intervention effectiveness for another public health issue).

| Evidence from level I–III study designs^a, b^ | Evidence from level IV studies^a^, indirect or parallel evidence [3] and/or from epidemiological modelling using a mixture of study designs |
| --- | --- |
| *1. ‘Sufficient evidence of effectiveness’* | *3. ‘Limited evidence of effectiveness’* |
| - The effect is unlikely to be due to chance or bias:   - evidence from a level I study design; several good quality level II studies; or several high quality level III-1 or III-2 studies from which effects of bias and confounding can be reasonably excluded. | - Sound theoretical rationale and programme logic; and - Level IV studies, indirect or parallel evidence for outcomes; or - Epidemiological modelling to the desired outcome (BMI) using a mix of evidence types or levels.   The effect is unlikely to be due to chance. Implementation of this intervention should be accompanied by an appropriate evaluation budget. |
| *2. ‘Limited evidence of effectiveness’* | *4. ‘Weak evidence of effectiveness’* |
| - The effect is probably not owing to chance. - Bias cannot be excluded as a possible explanation:   - evidence from one level II study of uncertain or indifferent quality; one level III-1 or III-2 study of high quality; several level III-1 or III-2 studies of lower quality; or sizeable number of level III-3 studies of good quality and consistent in suggesting an effect. | - Sound theoretical rationale and programme logic; or - Level IV studies, indirect or parallel evidence for outcomes; or - Epidemiological modelling to the desired outcome (BMI) using a mix of evidence types or levels.   The effect is probably not due to chance but bias cannot be excluded as a possible explanation.  Would benefit from further research and/or pilot studies before implementation. |
| *5. ‘Inconclusive evidence of effectiveness’* | *6. ‘No evidence of effectiveness’* |
| No position could be reached on the presence or absence of an effect of the intervention – only level III studies available but they are few and of poor quality. | No position could be reached on the likely credentials of this intervention. Further research may be warranted. |
| Abbreviations: BMI, body mass index. Boxes are numbered according to their order in the evidence hierarchy from 1 (strongest evidence) to 6 (no evidence). ^a^These evidence classifications are based on those of the National Health and Medical Research Council of Australia [4]: I, systematic review of RCTs; II, one or more properly designed RCTs; III, studies with other (non-randomized) controls; IV, case series, pre-test and/or post-test. ^b^See Carter et al. [5] or Haby et al. [6] for full details for this column. | |

1. GP prescription

Over 80% of the Australian population visit a general practitioner (GP) each year, making general practice an ideal setting for physical activity intervention. A physical activity prescription can be a relatively quick and easy tool that a GP may use to encourage patients to increase their physical activity. The prescription sets out the patient’s current activity levels, activity goals and instructions on how to achieve them, styled in a standard drug prescription format.

Most randomised controlled trials of GP prescription programs have found no significant effect on physical activity levels [7-10]. Key exceptions, however, are a randomised controlled trial of the STEP physical activity counselling program in Canada [11], which found that counselling with the addition of a prescription was more effective than counselling alone in improving physical fitness of sedentary older adults, and two randomised controlled trials of a Green Prescription program in New Zealand, in which patients receive additional follow-up support from exercise physiologists [12,13].

The randomised controlled trials could not be combined in a meta-analysis, due to different measures of physical activity and intervention characteristics (e.g. resource use). We selected the randomised controlled trial comparing New Zealand’s Green Prescription program with current practice [13] as the basis for cost-effectiveness analysis, because it had shown a significant effect on behaviour in more than one trial [12-14], the intervention had been compared with a ‘usual care’ control group, changes in behaviour had been followed up for at least one year, and the intervention could easily be adapted for the Australian health care system.

In the Green Prescription program, patients are screened opportunistically when they present for an appointment with a GP or PN. Inactive patients are provided with a written prescription by the GP or PN, and a copy of the patient information is faxed to an exercise physiologist who provides follow-up support by phone and mail-out for the first three months. Patients are also opportunistically followed up by the GP or PN.

Intervention effect

The mean difference in leisure-time energy expenditure between the Green Prescription intervention and control groups was 2.67 kcal/kg/wk (95% confidence interval: 0.48–4.86 kcal/kg/wk) at 12 months after the intervention [13].

Target group

The target population for analysis of the Green Prescription program is the Australian population of inactive adults aged between 40 and 79 years in 2003. Inactivity is defined as less than 30 minutes of moderate or vigorous activity on at least five days per week, and is determined from responses to questions on the duration and frequency of physical activity in Australia’s National Health Survey 2001 [15].

The annual average number of patients per GP is estimated, by age and sex, from the number of patients who visited a GP in 2002 [16], adjusted to the 2003 estimated resident population, and the number of full-time workload equivalent GPs in 2003-4 [17]. The number of GPs willing to participate in the intervention program is estimated from the total number of full-time workload equivalent GPs in 2003 [17], the mean rate of practice participation in the New Zealand trial of Green Prescription [13], and the average number of GPs per practice, which was estimated from BEACH (Bettering the Evaluation and Care of Health) data [18].

Rates of patient exclusion from the Australian Green Prescription program, either before or during the screening process, due to acute injury or illness, patient disinclination, existing medical conditions (e.g. unstable cardiac conditions), lack of English language skills or intent to leave the area, are assumed to be similar to those in the New Zealand trial [13]. Follow-up of patients who receive the intervention and patient completion of the intervention at twelve months are also assumed to occur at rates similar to those seen in the New Zealand trial [13].

Intervention costs

The Green Prescription intervention is delivered opportunistically (i.e. when the patient is visiting a GP or PN for another reason), thus the costs of delivery and follow-up are estimated as a proportion of the total consultation cost based on the number of sessions and length of sessions recorded by Elley et al. [19]. Costs of GP delivery and follow-up are derived from the Medicare Benefits Schedule for November 2003 [20]. Costs of PN delivery and follow-up are derived from the Second Practice Nurse Salary and Conditions Survey [21], with a 30% loading added to cover salary on-costs, such as Workcover, annual leave and superannuation.

In estimating time costs, the length of training sessions for GPs or PNs, the length of intervention delivery and follow-up sessions and number of sessions, and the length and number of phone calls between exercise physiologists and patients, are derived from results of the Green Prescription trial [13]. The cost of GP time in training to deliver the Green Prescription intervention is valued at an hourly wage rate evaluated by the Inner Eastern Melbourne Division of General Practice in 2001, and the cost of PN time for training is valued using the mean hourly wage rate from the Second Practice Nurse Salary and Conditions Survey [21]. The cost of patient time is valued from the *Average Weekly Earnings* [22], using labour force statistics from *Persons not in the labour force 2003* [23] and *Labour Force Australia 2003* [24].

Other costs associated with intervention set-up, training and co-ordination, and costs associated with providing exercise physiologist support to patients, including staff costs and overhead costs, are derived from the cost-effectiveness analysis of the Green Prescription program in New Zealand [19]. All costs are converted to Australian dollars using the Purchasing Power Parities [25] and adjusted to the year 2003 using the Health Price Index [26], where necessary.

1. GP referral to an exercise physiologist

For general practitioners, lack of time [27] and lack of confidence [28] are key barriers to delivery of physical activity advice during patient consultations. In a survey of West Australian GPs, 47% agreed or strongly agreed that referral to an exercise physiologist would be desirable practice [28]. Exercise physiologists may be better positioned to provide the expertise, time, monitoring and support for long-term behavioural change [29].

However, there have been few randomised controlled trials of GP referral to an exercise physiologist and the effects on physical activity behaviour are unclear. A randomised controlled trial in two general practices in Adelaide, South Australia, found that referral to an exercise physiologist led to a significant increase in the frequency and duration of physical activity after twelve months compared to a control group who did not receive the intervention [30]. However, two randomised controlled trials of a similar model of GP referral to an exercise physiologist in the UK did not find significant effects on physical activity behaviour [31,32].

Our cost-effectiveness analysis of GP referral to an exercise physiologist is based on the randomised controlled trial comparing referral with a no physical activity intervention control group in Adelaide [30][[2]](#footnote-3). In this intervention, patients aged 60 years or over were invited in a letter from their GP to attend a screening session at their local general practice. Sedentary patients who were enrolled in the intervention attended a session with an exercise physiologist where they were provided with individualised advice about the benefits of physical activity and discussed a pamphlet with a three-month plan of activity. Follow-up sessions were held with the exercise physiologist at three months and six months. All sessions with the exercise physiologist were held at the patients’ local GP practices, and participants were followed up in an interview held at the end of the trial at 12 months.

Intervention effect

Change in energy expenditure due to GP referral to an exercise physiologist is calculated by combining the number of sessions per week and minutes per session of walking and vigorous activity (Table II), assuming energy intensities of 3.5 METS for walking and 5.0 METS for more vigorous activity.

Table II: The number of sessions per week and minutes per session of walking and vigorous activity reported in the GP referral intervention and control groups at baseline and at 12 months [30]

|  | Intervention (n = 149) | | Control (n = 150) | |
| --- | --- | --- | --- | --- |
|  | **Baseline** | **12 months** | **Baseline** | **12 months** |
| Walking |  |  |  |  |
| Sessions/week* | 0 (0 – 1) | 3 (1 – 4) | 0 (0 – 2) | 2 (1 – 3) |
| Minutes/session | 0 (0 – 25) | 30 (10 – 60) | 0 (0 – 20) | 30 (10 – 60) |
| Vigorous activity |  |  |  |  |
| Sessions/week* | 0 (0 – 0) | 2 (0 – 3) | 0 (0 – 0) | 0 (0 – 1) |
| Minutes/session* | 0 (0 – 0) | 20 (0 – 35) | 0 (0 – 0) | 0 (0 – 15) |
| * p<0.05  NB. Values shown are median and 25^th^ and 75^th^ percentiles | | | | |

Target group

The target population for GP referral intervention is the Australian population of inactive adults aged at least 60 years in 2003, where inactivity is defined as not walking or taking brisk exercise for at least 20 minutes on at least three occasions per week. Rates of inactivity are determined from responses to questions on the duration and frequency of physical activity in Australia’s National Health Survey 2001 [15].

The annual average number of patients visiting a GP is estimated, by age and sex, from the number of patients who visited a GP in 2002 [16], adjusted to the 2003 estimated resident population, and the number of full-time workload equivalent GPs in 2003-4 [17]. The number of GPs willing to participate in the intervention program is estimated from the total number of full-time workload equivalent GPs in 2003 [17], Medicare statistics on GP participation in Practice Incentive Programs, and the average number of GPs per practice, which is estimated from BEACH (Bettering the Evaluation and Care of Health) data [18].

Rates at which patients attended screening sessions or returned screening questionnaires, as well as rates of medical exclusion, attendance at exercise physiologist consultations and completion of referral interventions are assumed to be similar to those observed in the randomised controlled trials.

Intervention costs

It is assumed that delivery would be co-ordinated through the Divisions of General Practice. Central co-ordination of the intervention would be handled by one full-time designated project officer in each State office and one half-time designated project officer in each Territory office; equivalent to seven project officers working on the intervention full-time for one year. It is assumed that the recruitment of GPs, patients and exercise physiologists, and routine monitoring of the program would be managed by project officers based in the local offices of the Divisions of General Practice. Allowance is made for one project officer in each of the 119 local Divisions of General Practice, working three days per week for the first eight weeks, during the intense period of GP recruitment and patient screening, and one day per week for the remaining weeks of the year, to facilitate communications between GPs, exercise physiologists and patients, and carry out routine monitoring of the program. Project officer salaries are estimated from an Australian Divisions of General Practice advertisement for a Youth mental health project officer in 2006. The assistance of study nurses in patient screening is costed at a casual rate for a Level One nurse of at least five years experience (Nurses (Queensland Public Health Sector) Award 2004).

Salaries for exercise physiologists are costed at the award rate for a Level Two/Three Professional Officer with at least four years experience (QLD Health Professional Stream Wage Rates).

Costs of production and delivery of the patient screening questionnaire are estimated from the per unit cost of printing and file processing of an A4 colour booklet (The Online Printer; www.theonlineprinter.com.au/quote_dis_booka4.aspx) and standard Australia Post rates. Costs of phone calls, letters and visits to GPs by project officers, mainly during the patient recruitment phase, are not costed separately, but are accounted for within a 60% loading on the salaries of all project officers, study nurses and exercise physiologists.

GP time in screening is valued at an hourly wage rate that was evaluated by the Inner Eastern Melbourne Division of General Practice in 2001. Each GP is assumed to spend a total of two hours in providing patient age-sex registers to project officers and sending letters to patients.

The length of screening sessions and intervention consultations are estimated from the outcomes of the GP referral trial [30,33]. In keeping with the ACE economic protocol [34], it is assumed that each patient would spend a total of 30 minutes in travelling to and from their appointment and spend an average of 15 minutes waiting to see the exercise physiologist.

Costs of patient travel to and from screening and intervention sessions are included. Travel distance is estimated from reports of average distances travelled to visit GPs in regional [35] and remote [36] areas and estimates of urban travel distances. Vehicle operating costs are estimated from the 2003 Royal Automobile Club Victoria private vehicle reimbursement rates for medium 2-3 litre vehicles.

1. Mass media-based community campaign

**Mass media interventions use the power of television, radio, newspapers, internet, billboards and other forms of mass communication to deliver health messages. Mass media is often integrated with community events and activities, and distribution of promotional material, as part of a community-wide campaign.**

**There is good evidence that these types of campaigns can raise awareness of physical activity health benefits and increase knowledge about physical activities [37]. A review of 20 mass media-based community campaigns found mixed outcomes of effect on physical activity behaviour, but concluded that on the basis of the short-term evidence available, these campaigns can have modest short-term impacts on behaviour [38].**

The cost-effectiveness analysis of mass media intervention for physical activity is modelled on a series of mass media-based community campaigns run in New South Wales in 1998 (six-week campaign targeting 25 to 60 year olds) and 1999 (four-week campaign targeting 55+ year olds). Each intervention included paid television and print media advertising, information for public health professionals and family physicians, community events and promotions, and marketing of campaign merchandise [39,40].

Intervention effect

Bauman et al [39] measured the effect of the six-week mass media intervention in New South Wales by telephone survey of independent populations before and after the campaign, both in the campaign state of New South Wales and in the rest of Australia, which did not receive the intervention. Physical activity in the past week was measured in total hours of activity (Table III) as well as by achievement of a physical activity threshold of five sessions and 150 minutes of activity. There was a non-significant increase in physical activity in the target age group of 25 to 60 year olds in the intervention state, but physical activity levels decreased significantly in the rest of Australia.

A later evaluation of the four-week campaign targeting 55+ year olds did not observe a significant change in physical activity behaviour in the population receiving the intervention [40]. However, no sampling was carried out in an unexposed population (e.g. in the rest of Australia) for comparison.

To model the intervention in the Australian population, an intervention effect is calculated in MET-minutes per week by taking the product of the net increase in total hours of physical activity per week (Table III), and the mean intensity of physical activity in 25 to 60 year olds, which is derived from National Health Survey data [41].

Table III: Effect of mass media campaign on total hours of physical activity in past week [39].

|  | **Pre-campaign**  Mean (SD) | **Post-campaign**  Mean (SD) |
| --- | --- | --- |
| New South Wales | 4.22 (4.7)  n = 2029 | 4.41 (4.8)  n = 1700 |
| Rest of Australia | 4.67 (4.98)  n = 3006 | 4.22 (4.53)*  n = 2253 |
| * p = 0.06 | | |

Target group

In the cost-effectiveness analysis, the intervention is targeted at all 25 to 60 year olds in the Australian population, the target age range for which an intervention effect has been observed. There were 9.74 million people in this age range in Australia in 2003 [42].

Intervention costs

Costs of an Australia-wide mass media intervention are derived from cost estimates for the NSW Health campaign in 1999. Key components include the cost of printing and developing materials (e.g. television commercial, brochures and posters), buying television media, community service announcements (radio announcements broadcast free of charge), supplementary grants for community development initiatives and tracking surveys [40].

All component costs were inflated to 2003 dollars using the Consumer Price Index [43]. Costs were scaled up, where relevant, to a magnitude associated with a six-week campaign targeting 9.74 million Australia-wide (rather than a four-week campaign targeting 1.4 million in New South Wales), then summed to determine the total cost of a mass media campaign intervention in Australia (Table IV).

Table IV: Mass media intervention costs.

| **Cost component** | **Cost**  **($ million)** | **Assumptions** |
| --- | --- | --- |
| Intervention material development | $0.23 | One-off cost; scaled from 4- to 6-week intervention. |
| Intervention material printing | $1.1 | Cost scaled to intervention target population of 9.74 million. |
| Television media buy | $3.1 | Cost scaled to 8 states/territories; scaled from 4- to 6-week intervention. |
| Supplementary grants for community development initiatives | $1.1 | Cost scaled to intervention target population of 9.74 million; scaled from 4- to 6-week intervention. |
| Tracking surveys | $0.34 | Cost scaled to intervention target population of 9.74 million. |
| Other (e.g. production of community service announcements) | $0.22 | Cost scaled to intervention target population of 9.74 million; scaled from 4- to 6-week intervention. |
| Community service announcements* | $7.2 | Cost scaled to 8 states/territories; scaled from 4- to 6-week intervention. |
| **Total cost** | **$13.3** |  |
| * Community service announcements were delivered free of charge in the 1999 NSW Health campaign, but are still included as part of the total intervention cost. | | |

1. Pedometer campaign

A pedometer is a small, lightweight device that can be worn at the hip to count the number of steps that are taken. Typically less than $50 per device[[3]](#footnote-4), the pedometer is a relatively low cost method of measuring physical activity in the form of walking or running.

An active lifestyle has been defined as a minimum of 10,000 steps per day [44]. In intervention trials, participants are asked to keep a daily record of the number of steps taken and may be given a specific step goal, such as 10,000 steps per day. The use of pedometers is associated with significant increases in physical activity [45].

Intervention effect

The effect of pedometer use is calculated in MET-minutes per week from the daily change in steps counted by the pedometer and an assumed step pace. Meta-analysis of eight randomised controlled trials of pedometer use, found that pedometer users significantly increased their activity by 2,491 steps per day (95% CI: 1,098 – 3,885 steps per day) more than control participants [45]. Average step pace for males and females is calculated to be 106 steps per minute, using regression equations defined by Tudor-Locke et al. [46], and assuming that the additional steps per day will be achieved at a walking intensity of 3.5 METS.

Target group

The pedometer intervention is modelled as a population-wide intervention; targeting everyone in the Australian population aged 15 and over in 2003. However, it is likely that only a small proportion of the target population will choose to participate in the intervention. In a two-year follow-up survey of an Australian pedometer intervention, the Rockhampton 10,000 steps program, 18.1% of respondents in the intervention community of Rockhampton reported using a pedometer in the previous 18 months compared to 5.6% of respondents in the comparison community of Mackay, which did not participate in the pedometer program [47]. Therefore, in the cost-effectiveness analysis of pedometer intervention in Australia, it is assumed that 12.5% (i.e. 18.1% – 5.6%) of the target population would start using a pedometer due to the intervention.

Intervention costs

The costs of running a pedometer intervention program include direct intervention costs and costs of project management, such as salaries for staff to implement and monitor the program.

The direct intervention costs are derived from the number of participants in the target group and a cost per participant. The cost per participant is evaluated as a weighted average of intervention costs from each randomised controlled trial in the meta-analysis of pedometer intervention effect [45]. Each randomised controlled trial intervention is costed from the quantities of resources used and unit cost of each resource component (Table V). Project management costs are then estimated proportionally, based on the reported split in funding for the Rockhampton 10,000 steps program, where direct intervention costs accounted for 70% of project funds [48].

1. TravelSmart active transport

Two-thirds of Australians live less than 20km from their place of work or study, yet only 6% of Australia’s workforce and students aged 18 years and older usually walk or cycle to their destination [49]. The aim of active transport intervention is to shift people from car-use to walking or cycling as a mode of transport.

Methods of active transport intervention include targeted behaviour change programs (e.g. visits to assenting households with tailored information and advice), publicity campaigns, engineering measures (e.g. building new bike paths), financial incentives and provision of alternative transport services. A systematic review of these interventions found that the targeted behaviour change programs can be effective in shifting people towards active transport modes, but evidence for other interventions was not yet of sufficient rigour to establish effectiveness [50]. The cost-effectiveness analysis, therefore, focuses on targeted behaviour change.

Table V: Estimates of resource use and cost per participant of pedometer intervention.

|  |  | **RESOURCE USE** | | | | **COSTS (AUS$2003)** | | | | |
| --- | --- | --- | --- | --- | --- | --- | --- | --- | --- | --- |
| **Source** | **Intervention participants** | **Pedometers** | **Counselling** | **Information pack** | **Telephone calls** | **Pedometers** | **Counselling** | **Information pack** | **Telephone calls** | **Total** |
| Araiza et al, (2006)[51] | 15 | 1 | – | – | – | $41.44 | – | – | – | $41.44 |
| Butler and Dwyer, (2004)[52] | 17 | 1 | – | – | – | $41.44 | – | – | – | $41.44 |
| de Blok et al, (2006)[53] | 10 | 1 | 4 × 30 mins  Physiotherapist | – | – | $41.44 | $201.32 | – | – | $242.76 |
| Hultquist et al, (2005)[54] | 31 | 1 | – | – | – | $41.44 | – | – | – | $41.44 |
| Izawa et al, (2005)[55] | 25 | 1 | – | – | – | $41.44 | – | – | – | $41.44 |
| Moreau et al, (2001)[56] | 15 | 1 | – | – | – | $41.44 | – | – | – | $41.44 |
| Ransdell et al, (2004)[57]; Ornes et al, (2005)[58] | 30 | 1 | 2 × 120 mins  Exercise physiologist* | 1 | 6 × 3 mins**  Exercise physiologist* | $41.44 | $106.05 | $0.93 | $7.33 | $155.75 |
| Talbot et al, (2003)[59] | 17 | 1 | 12 × 5 mins  Registered nurse | 1 | – | $41.44 | $25.31 | $0.50 | – | $67.26 |
| * Provider assumed to be exercise physiologist.  ** Average telephone call assumed to last 3 minutes.  *** Weighted by number of participants in each study. | | | | | | | | | **Weighted average***** | **$78.20** |

In Australia, Germany, Austria, France, Sweden, Switzerland, the United Kingdom and the United States, targeted behaviour change has been implemented in the form of TravelSmart (or ‘Indimark’) [60]. In this program, households are recruited via telephone and/or mail, and assenting households are classified according to travel behaviour and interests. Households already meeting active travel goals are rewarded with local information and/or merchandise (e.g. water bottles, key rings), and those who are interested in active travel are visited and also provided with tailored information (e.g. maps of local walking trails) and merchandise. Local transport operators and councils are typically involved in providing timetables, maps and other information.

Intervention effect

The primary focus of the TravelSmart approach to personalised marketing is improving environmental sustainability through reducing car use. Evidence of intervention effectiveness is chiefly located in consultant reports to governments, with a few studies reported in transport-related conferences and journals. Only English language sources were evaluated.

Studies were located for personalised marketing in 37 cities around the world. However, only 20 cities had sufficient data to evaluate an intervention health effect – ten in Australia [61-70], six in the United Kingdom [71-75], three in the United States [76] and one in Germany [77].

Each study involved a telephone survey of independent community samples before and after the intervention. An increase in physical activity time is derived from the change in trips per person per year, either walking or by bicycle, and the average minutes per trip by each mode of transport. For eight studies that do not report trip times, the minutes per trip for each mode are estimated as the weighted average of the other eleven studies that did report trip times.

The increase in physical activity time is combined with activity intensity (3.5 METS for walking and 5 METS for bicycling) to determine the average change in energy expenditure due to the intervention. It is assumed that the increase in time spent in active transport is not counteracted by a reduction in other activity (e.g. leisure time activity).

Target group

The active transport intervention is targeted at households in urban areas of Australia. With 75% of the Australian population living in urban areas [78], 2.5 people per household [79], and an average of 91% of households able to be contacted in previous individualised marketing studies [60-77,80-82], a total of 5.4 million households would receive the intervention.

Intervention costs

The interventions costs are derived from the annual reported cost of delivering the personalised marketing intervention in Western Australia between 2002 and 2007 [83-87]. When adjusted for CPI [43], the mean cost to deliver the intervention is $83 (95%CI: $62 – $104) per household.

1. Internet-based intervention

Intervention delivery across the internet, via websites, chat rooms and/or email, is a relatively new mode of physical activity intervention. Internet users are generally recruited via newspaper advertisements, flyers, television or email, and the participants receive information and/or participate in consultations without face-to-face contact.

Potential advantages of internet-based intervention include low cost and a high level of accessibility [88], but the design and delivery of internet interventions and evaluation of their effect on physical activity behaviour is still at an early stage of development. A systematic review of randomised controlled trials and quasi-experimental studies of internet-delivered physical activity intervention targeting adults reported improvements in physical activity in eight out of fifteen studies [89].

Intervention effect

Three of the fifteen studies identified in the systematic review [89] compared internet intervention targeting physical activity behaviour with a no intervention control group that is comparable to current practice in Australia. The intervention effect was calculated by meta-analysis of these three studies [90-92], using STATA (Version 8), but results are heavily influenced by one much larger study [92].

Target group

The intervention is targeted at the Australian population aged 15 years and older. There are, on average, 2.5 people per household [79], and 63% of households have internet access [93]. It is assumed that 23% of this population could be made aware of the internet intervention through the media, based on the rates of unprompted message recall in a post-physical activity media campaign telephone survey in New South Wales [39].

Rates of intervention participation (17%) and attrition (18%) are derived as a weighted average of the rates in the three studies included in the meta-analysis of effect.

Intervention costs

Costs associated with an internet intervention can include costs of establishing a website (e.g. hardware, software, service provider, domain name), developing the intervention (e.g. intellectual property, software), operating the website (e.g. service provider fees, web maintenance, security) and recruiting participants (e.g. newspaper advertising, search engine marketing, email marketing). The cost of the internet intervention for physical activity is based on the annual cost of providing a Government health information website in Victoria. This was provided at a mean cost of $21 million (range: $2 million to $44 million) between 2001-02 and 2004-05 [94-97].

References

1. ABS (2001) National Health Survey: Users' Guide. Canberra: Australian Bureau of Statistics.

2. Haby M, Vos T, Carter R, Moodie M, Markwick A, et al. (2006) A new approach to assessing the health benefit from obesity interventions in children and adolescents: the Assessing Cost-Effectiveness in obesity project. International Journal of Obesity 30: 1463-1475.

3. Swinburn B, Gill T, Kumanyika S (2005) Obesity prevention: a proposed framework for translating evidence into action. Obesity Reviews 6: 23-33.

4. NHMRC (1999) A guide to the development, implementation and evaluation of clinical practice guidelines. Canberra: National Health and Medical Research Council.

5. Carter R, Stone C, Vos T, Hocking J, Mihalopoulos C, et al. (2000) Trial of program budgeting and marginal analysis (PBMA) to assist cancer control planning in Australia. Melbourne: Centre for Health Program Evaluation. Research report 19 Research report 19.

6. Haby MM, Carter R, Mihalopoulos C, Magnus A, Sanderson K, et al. (2004) Assessing cost-effectiveness - Mental health: introduction to the study and methods. Australian and New Zealand Journal of Psychiatry 38: 569-578.

7. Harland J, White M, Drinkwater C, Chinn D, Farr L, et al. (1999) The Newcastle exercise project: a randomised controlled trial of methods to promote physical activity in primary care. BMJ 319: 828-832.

8. Goldstein MG, Whitlock EP, DePue J (2004) Multiple behavioral risk factor interventions in primary care: Summary of research evidence. American Journal of Preventive Medicine 27: 61-79.

9. Simons-Morton D (2001) Effects of Physical Activity Counseling in Primary Care: The Activity Counseling Trial: A Randomized Controlled Trial. JAMA 286: 677-687.

10. Hillsdon M, Thorogood M, White I, Foster C (2002) Advising people to take more exercise is ineffective: a randomized controlled trial of physical activity promotion in primary care. International Journal of Epidemiology 31: 808-815.

11. Petrella RJ, Koval JJ, Cunningham DA, Paterson DH (2003) Can primary care doctors prescribe exercise to improve fitness?: The step test exercise prescription (STEP) project. American Journal of Preventive Medicine 24: 316-322.

12. Swinburn BA, Walter LG, Arroll B, Tilyard MW, Russell DG (1998) The green prescription study: A randomized, controlled trial of written exercise advice provided by general practitioners. American Journal of Public Health 88: 288-291.

13. Elley CR, Kerse N, Arroll B, Robinson E (2003) Effectiveness of counselling patients on physical activity in general practice: cluster randomised controlled trial. BMJ 326: 793-796.

14. Lawton BA, Rose SB, Elley CR, Dowell AC, Fenton A, et al. (2008) Exercise on prescription for women aged 40-74 recruited through primary care: two year randomised controlled trial

10.1136/bmj.a2509. BMJ 337: a2509-.

15. ABS (2001) National Health Survey, Confidentialised Unit Record File, Australia on CD Rom , 2001. Canberra: Australian Bureau of Statistics.

16. Beilby J, Furler J (2005) General practitioner services in Australia. General Practice in Australia: 2004: Commonwealth of Australia. pp. 128-213.

17. Department of Health and Ageing (2006) General Practice Statistics: Number of General Practitioners. Australian Government Department of Health and Ageing.http://www.health.gov.au/internet/wcms/publishing.nsf/Content/health-pcd-statistics-gpnos.htm

18. Britt H, Miller G, Knox S, Charles J, Valenti L, et al. (2004) General practice activity in Australia 2003–04. Canberra: Australian Institute of Health and Welfare.

19. Elley CR, Kerse N, Arroll B, Swinburn B, Ashton T, et al. (2004) Cost effectiveness of physical activity counselling in general practice. NZMJ 117: U1216.

20. Department of Health and Ageing (2003) Medicare Benefits Schedule. Canberra: Commonwealth of Australia.

21. Australian Practice Nurse Association (2006) 2006 APNA Practice Nursing Salary and Conditions Survey.www.apna.asn.au/client_images/51288.pdf

22. ABS (2003) Average Weekly Earnings. Canberra: Australian Bureau of Statistics.

23. ABS (2003) Persons not in the labour force 2003. Canberra: Australian Bureau of Statistics.

24. ABS (2003) Labour Force Australia 2003. Canberra: Australian Bureau of Statistics.

25. OECD (2006) Purchasing Power Parities (PPPs) for OECD Countries 1980-2006. Organisation for Economic Co-operation and Development.www.oecd.org/std/ppp/

26. AIHW (2006) Health expenditure Australia. Canberra: Australian Institute of Health and Welfare.

27. Eakin E, Smith B, Bauman A (2005) Evaluating the population health impact of physical activity interventions in primary care - are we asking the right questions? Journal of Physical Activity and Health 2: 197-215.

28. Bull FCL, Schipper ECC, Jamrozik K, Blanksby BA (1997) How Can and Do Australian Doctors Promote Physical Activity? Preventive Medicine 26: 866-873.

29. Handcock P, Jenkins C (2003) The green prescription: a field of dreams? New Zealand Medical Journal 116: 1187.

30. Halbert JA, Silagy CA, Finucane PM, Withers RT, Hamdorf PA (2000) Physical activity and cardiovascular risk factors: effect of advice from an exercise specialist in Australian general practice. Medical Journal of Australia 173: 84-87.

31. Stevens W, Hillsdon M, Thorogood M, McArdle D (1998) Cost-effectiveness of a primary care based physical activity intervention in 45-74 year old men and women: a randomised controlled trial. British Journal of Sports Medicine 32: 236-241.

32. Harrison RA, Roberts C, Elton PJ (2005) Does primary care referral to an exercise programme increase physical activity 1 year later? A randomized controlled trial. Journal of Public Health 27: 25-32.

33. Halbert JA, Silagy CA, Finucane P, Withers RT, Hamdorf PA (1999) Recruitment of older adults for a randomized, controlled trial of exercise advice in a general practice setting. Journal of the American Geriatrics Society 47: 477-481.

34. Carter R, Vos T, Moodie M, Haby M, Magnus A, et al. (Accepted) Priority Setting in Health: Origins, Description and Application of the Assessing Cost Effectiveness (ACE) Initiative. Expert Review of Pharmacoeconomics and Outcomes Research.

35. Rankin S, Hughes-Anderson W, House J, Aitken J, Heath D, et al. (2002) Rural residents' utilisation of health and visiting specialist health services. Rural and Remote Health 2: 119.

36. Bamford E, Dunne L, Taylor D, Symon B, Hugo G, et al. (1999) Accessibility to general practitioners in rural South Australia. A case study using geographic information system technology. Medical Journal of Australia 171: 614-616.

37. Kahn EB, Ramsey LT, Brownson RC, Heath GW, Howze EH, et al. (2002) The effectiveness of interventions to increase physical activity: A systematic review,. American Journal of Preventive Medicine 22: 73-107.

38. Marshall AL, Owen N, Bauman AE (2004) Mediated approaches for influencing physical activity: update of the evidence on mass media, print, telephone and website delivery of interventions. Journal of Science and Medicine in Sport 7: 74-80.

39. Bauman AE, Bellew B, Owen N, Vita P (2001) Impact of an Australian mass media campaign targeting physical activity in 1998. American Journal of Preventive Medicine 21: 41-47.

40. NSW Health (2000) Public education campaign to promote physical activity among older people: NSW evaluation report. NSW Department of Health.

41. ABS (2005) National Health Survey - Confidential Unit Record Files, 2004-05. Canberra: Australian Bureau of Statistics.

42. ABS (2004) Population by Age and Sex, Australian States and Territories, June 2004. Canberra: Australian Bureau of Statistics.

43. ABS (2006) Consumer Price Index, Australia, June quarter. Canberra: Australian Bureau of Statistics.

44. Tudor-Locke C, Basset Jr D (2004) How Many Steps/Day Are Enough? Preliminary Pedometer Indices for Public Health. Sports Medicine 34: 4-8.

45. Bravata DM, Smith-Spangler C, Sundaram V, Gienger AL, Lin N, et al. (2007) Using pedometers to increase physical activity and improve health - A systematic review. Jama-Journal of the American Medical Association 298: 2296-2304.

46. Tudor-Locke C, Sisson SB, Collova T, Lee SM, Swan PD (2005) Pedometer-determined step count guidelines for classifying walking intensity in a young ostensibly healthy population. Canadian Journal of Applied Physiology-Revue Canadienne De Physiologie Appliquee 30: 666-676.

47. Eakin EG, Mummery K, Reeves MM, Lawler SP, Schofield G, et al. (2007) Correlates of pedometer use: Results from a community-based physical activity intervention trial (10,000 Steps Rockhampton). International Journal of Behavioral Nutrition and Physical Activity 4: 31.

48. Brown W, Mummery W, Eakin E, Schofield G (2006) 10,000 Steps Rockhampton: evaluation of a whole community approach to improving population levels of physical activity. Journal of Physical Activity and Health 3: 1-15.

49. ABS (2006) Environmental Issues, People's Views and Practices, Australia. Canberra: Australian Bureau of Statistics.

50. Ogilvie D, Egan M, Hamilton V, Petticrew M (2004) Promoting walking and cycling as an alternative to using cars: systematic review. British Medical Journal 329: 763-766B.

51. Araiza P, Hewes H, Gashetewa C, Vella CA, Burge MR (2006) Efficacy of a pedometer-based physical activity program on parameters of diabetes control in type 2 diabetes mellitus. Metabolism-Clinical and Experimental 55: 1382-1387.

52. Butler L, Dwyer D (2004) Pedometers may not provide a positive effect on walking activity. Health Promotion Journal of Australia 15: 134-136.

53. de Blok BMJ, de Greef MHG, ten Hacken NHT, Sprenger SR, Postema K, et al. (2006) The effects of a lifestyle physical activity counseling program with feedback of a pedometer during pulmonary rehabilitation in patients with COPD: A pilot study. Patient Education and Counseling 61: 48-55.

54. Hultquist CN, Albright C, Thompson DL (2005) Comparison of walking recommendations in previously inactive women. Medicine and Science in Sports and Exercise 37: 676-683.

55. Izawa KP, Watanabe S, Onvya K, Hirano Y, Oka K, et al. (2005) Effect of the self-monitoring approach on exercise maintenance during cardiac rehabilitation - A randomized, controlled trial. American Journal of Physical Medicine & Rehabilitation 84: 313-321.

56. Moreau KL, Degarmo R, Langley J, McMahon C, Howley ET, et al. (2001) Increasing daily walking lowers blood pressure in postmenopausal women. Medicine and Science in Sports and Exercise 33: 1825-1831.

57. Ransdell LB, Robertson L, Ornes L, Moyer-Mileur L (2004) Generations exercising together to improve fitness (GET FIT): A pilot study designed to increase physical activity and improve health-related fitness in three generations of women. Women & Health 40: 77-94.

58. Ornes LL, Ransdell LB, Robertson L, Trunnell E, Moyer-Mileur L (2005) A 6-month pilot study of effects of a physical activity intervention on life satisfaction with a sample of three generations of women. PERCEPTUAL AND MOTOR SKILLS 100: 579-591.

59. Talbot LA, Gaines JM, Huynh TN, Metter EJ (2003) A home-based pedometer-driven walking program to increase physical activity in older adults with osteoarthritis of the knee: A preliminary study. Journal of the American Geriatrics Society 51: 387-392.

60. Brog W, Erl E, Mense N (2002) Individualised Marketing: Changing Travel Behaviour for a better Environment. OECD Workshop: Environmentally Sustainable Transport. 5 - 6 December 2002, Berlin.

61. Socialdata Australia (2003) Final Report: TravelSmart® Households - Marangaroo. Western Australia: Department for Planning and Infrastructure.

62. Socialdata Australia (2003) Final Report: TravelSmart® Households - Cambridge. Western Australia: Department for Planning and Infrastructure.

63. Socialdata Australia (2004) Final Report: TravelSmart® Households - City of Subiaco. Western Australia: Department for Planning and Infrastructure.

64. Socialdata Australia (2004) Final Report: TravelSmart® Households - City of Freemantle. Western Australia: Department for Planning and Infrastructure.

65. Socialdata Australia, Institute for Transport and Infrastructure Research Ltd (2004) Final Report: TravelSmart® Darebin. Victoria: Department for Infrastructure.

66. Socialdata Australia, Institute for Transport and Infrastructure Research Ltd (2004) Final Report: TravelSmart® Alamein. Victoria: Department for Infrastructure.

67. Socialdata Australia (2005) Final Report: TravelSmart® Households - City of Belmont. Western Australia: Department for Planning and Infrastructure.

68. Socialdata Australia (2005) Final Report: TravelSmart® Households - Town of Vincent. Western Australia: Department for Planning and Infrastructure.

69. Socialdata Australia (2005) Final Report: TravelSmart® Households - City of Armadale. Western Australia: Department for Planning and Infrastructure.

70. Socialdata Australia (2007) TravelSmart® Household Final Evaluation Report: City of Wanneroo - Clarkson and Surrounds. Western Australia: Department for Planning and Infrastructure.

71. Sustrans (2004) TravelSmart Bristol (Bishopston). UK: TravelSmart.

72. Sustrans (2004) TravelSmart Gloucester (Quedgeley). UK: TravelSmart.

73. Sustrans (2004) TravelSmart Sheffield (Hillsborough/Middlewood) 2003-04. UK: TravelSmart.

74. Sustrans (2004) Greater Nottingham TravelSmart (Lady Bay and the Meadows). UK: TravelSmart.

75. Sustrans (2004) TravelSmart Northumberland (Cramlington). UK: TravelSmart.

76. Socialdata America (2004) IndiMark® and Behavior Change Results: For the Cities of Salem-Keizer, Eugene, and Bend, Oregon. Oregon Department of Transportation.

77. Tapestry (2003) Viernheim Household Transport.www.max-success.eu/tapestry/www.eu-tapestry.org/p_dwl/csr/csr_c6_germany.pdf

78. ABS (2004) Population by Age and Sex, Australia - Electronic Delivery, Jun 2004. Canberra: Australian Bureau of Statistics.

79. ABS (2007) Year Book Australia, 2007. Canberra: Australian Bureau of Statistics.

80. Marinelli P, Roth M (2002) Behaviour change sustainability from Individualised Marketing. 25th Australasian Transport Research Forum. 2 - 4 October 2002, Canberra.

81. James B, Brog W, Erl E, Funke S (1999) Behaviour change sustainability from Individualised Marketing. 23rd Australasian Transport Research Forum. 29 September - 1 October 1999, Perth, Western Australia.

82. Socialdata Australia (2004) Final Report: TravelSmart® Households - City of Melville. Western Australia: Department for Planning and Infrastructure.

83. DPI (2003) Annual Report 2002-03. Department for Planning and Infrastructure, Government of Western Australia.

84. DPI (2004) Annual Report 2003-04. Department for Planning and Infrastructure, Government of Western Australia.

85. DPI (2005) Annual Report 2004-05. Department for Planning and Infrastructure, Government of Western Australia.

86. DPI (2006) Annual Report 2005-06. Department for Planning and Infrastructure, Government of Western Australia.

87. DPI (2007) Annual Report 2006-07. Department for Planning and Infrastructure, Government of Western Australia.

88. Ritterband L, Gonder-Frederick L, Cox D, Clifton A, West R, et al. (2003) Internet Interventions: In Review, In Use, and Into the Future. Professional Psychology: Research and Practice 34: 527-534.

89. Vandelanotte C, Spathonis KM, Eakin EG, Owen N (2007) Website-Delivered Physical Activity Interventions: A Review of the Literature. American Journal of Preventive Medicine 33: 54-64.

90. Kosma M, Cardinal BJ, McCubbin JA (2005) Longitudinal effects of a web-based physical activity motivational program among adults with physical disabilities. Research Quarterly for Exercise and Sport 76: A116-A116.

91. Napolitano MA, Fotheringham M, Tate D, Sciamanna C, Leslie E, et al. (2003) Evaluation of an Internet-based physical activity intervention: A preliminary investigation. Annals of Behavioral Medicine 25: 92-99.

92. Plotnikoff RC, McCargar LJ, Wilson PM, Loucaides CA (2005) Efficacy of an e-mail intervention for the promotion of physical activity and nutrition behavior in the workplace context. American Journal of Health Promotion 19: 422-429.

93. ABS (2006) Patterns of internet access in Australia. Canberra: Australian Bureau of Statistics.

94. Department of Human Services (2002) Annual Report 2001-02. Victorian Government.

95. Department of Human Services (2003) Annual Report 2002-03. Victorian Government.

96. Department of Human Services (2004) Annual Report 2003-04. Victorian Government.

97. Department of Human Services (2005) Annual Report 2004-05. Victorian Government.

1. A MET (metabolic equivalent) is the ratio of the working metabolic rate to the resting metabolic rate (1 MET = 1 kcal/kg/hr) [↑](#footnote-ref-2)
2. We found no significant difference in cost-effectiveness of the Adelaide and UK interventions in preliminary analyses; the results of the Adelaide intervention are included in this study because the intervention would not require any adaption for delivery in general practice in Australia. [↑](#footnote-ref-3)
3. A range of pedometer costs can be viewed at: http://www.healthmg.com.au/type.idc?Ptype=Pedometers&start=0 [↑](#footnote-ref-4)
