# Supplementary material for: Cost-Effectiveness of Interventions to Promote Physical Activity: A Modelling Study
Source: PLoS Med. 2009 Jul 14;6(7):e1000110. doi: 10.1371/journal.pmed.1000110 (PMC2700960; doi:10.1371/journal.pmed.1000110)
Supplement: Text S2 — Cost-effectiveness modelling methods. (0.16 MB DOC) [file pmed.1000110.s002.doc]

**Cost-effectiveness modelling methods**

A model was developed to evaluate the cost-effectiveness of physical activity interventions. The model evaluates intervention cost-effectiveness over the lifetime of the Australian population in a baseline year of 2003, using information on intervention costs and effects from the physical activity intervention literature and Australian data on disease epidemiology and costs of disease treatment. The model has been built in Excel (Microsoft Office 2003) and uses the add-in tool @Risk (Palisade, Version 4.5) for uncertainty analysis.

**Life table analysis**

In a proportional multi-state lifetable [1] (Figure I), disability-adjusted life years (DALYs) averted by an intervention are evaluated as the difference in health-adjusted years of life lived between an Australian population that continues to be physically active at current rates and an identical population that receives the intervention. The health-adjusted years of life lived by each population are calculated by dividing each population into five-year age group cohorts (from age 15-19 to age 95+), and simulating each cohort in a life table until everyone has either died or reached 100 years of age.

Years of life lived by each cohort are adjusted at each age for time spent in poor health (‘disability’) due to disease or injury, using disability estimates for the Australian population [2]. For modelled disease, the disability adjustment is calculated as the number of prevalent years lived with disability (pYLD) per prevalent case of disease. The age- and sex-specific probability of health loss due to disability from all other causes (i.e. those not specifically modelled) is calculated as the number of pYLD per capita for these conditions.

Figure I: Schematic of a proportional multi-state lifetable, showing the interaction between disease parameters and lifetable parameters, where x is age, i is incidence, p is prevalence, m is mortality, w is disability-adjustment, q is probability of dying, l is number of survivors, L is life years, Lw is disability-adjusted life years and HALE is health-adjusted life expectancy, and where ‘-‘ denotes a parameter that specifically excludes modelled diseases, and ‘+’ denotes a parameter for all diseases (i.e. including modelled diseases).

**Disease models**

Physical inactivity increases risk of ischaemic heart disease, ischaemic stroke, type 2 diabetes, breast cancer and colon cancer [3]. Each of these diseases is modelled explicitly using a set of differential equations that describe the transition of people between four states (healthy, diseased, dead from the disease, and dead from all other causes), with transition of people between the four states based on rates of mortality, incidence, case fatality and remission (Figure II) [4].

Epidemiological data inputs to the disease models are derived from the Australian Burden of Disease study [2], with the aid of DISMOD to derive data not explicitly reported (e.g. case fatality and prevalence from incidence and mortality rates). Future changes in disease incidence and case fatality are estimated from the twenty year predictions in the Australian Burden of Disease study, with trends assumed to remain constant thereafter.

**Intervention effect on physical activity**

The health benefits of an intervention are modelled as a reduction in incidence of each physical activity-related disease. Intervention combinations are assumed to have a multiplicative effect (Equation 1).

Healthy

Diseased

Dead

(disease)

Dead

(other)

Mortality

(other)

Remission

Incidence

Case

fatality

Mortality (other)

Figure II: Each disease in the physical activity analysis is modelled by a conceptual model with four states (healthy, diseased, dead from the disease, and dead from all other causes) and transition hazards between states of incidence, remission, case fatality and mortality from all other causes (after Barendregt et al. [4])

(1)

where:

is the incidence of disease (e.g. ischaemic heart disease) in the population (by age and sex);

is the new incidence of disease (e.g. ischaemic heart disease) when an intervention is implemented; and

is the potential impact fraction (PIF) for interventions *1* to *n*.

Each potential impact fraction (PIF) is derived from three key parameters: the population prevalence of physical activity in Australia, the relative risks of physical activity-related diseases, and the change in energy expenditure due to an intervention (in MET-minutes per week). The physical activity prevalence is derived from the distribution of energy expenditure in the Australian population, which is determined from responses to questions on the type, frequency and duration of physical activity in the National Health Survey 2004-05 [5]. Physical activity is divided into three categories (Figure III), based on total weekly energy expenditure: inactive (<100 MET-minutes per week), insufficiently active (100 – 750 MET-minutes per week) and sufficiently active (≥ 750 MET-minutes per week). Relative risks of disease in each physical activity category (Table I) are drawn from meta-analyses carried out for the global *Comparative Quantification of Health Risks* [3]. Since type 2 diabetes is itself a risk factor for developing cardiovascular disease, hazard ratios from the Asia Pacific Cohort Study Collaboration [6] are used to calculate the risk of ischaemic heart disease and stroke in those with type 2 diabetes, while constraining the total risk in the diabetic and non-diabetic population to the published risks of ischaemic heart disease and stroke.

Table I: Relative risks of disease due to physical inactivity [3]

|  | **Age** | **Inactive** | **Insufficient** | **Sufficient** |
| --- | --- | --- | --- | --- |
| Ischaemic heart disease* | 15-69  70-79  80+ | 1.71 (1.58-1.85)  1.50 (1.38-1.61)  1.31 (1.21-1.41) | 1.44 (1.28-1.62)  1.31 (1.17-1.48)  1.20 (1.07-1.35) | 1.00  1.00  1.00 |
| Ischaemic stroke* | 15-69  70-79  80+ | 1.53 (1.31-1.79)  1.38 (1.18-1.60)  1.24 (1.06-1.45) | 1.10 (0.89-1.37)  1.08 (0.87-1.33)  1.05 (0.85-1.30) | 1.00  1.00  1.00 |
| Type 2 diabetes | 15-69  70-79  80+ | 1.45 (1.37-1.54)  1.32 (1.25-1.40)  1.20 (1.14-1.28) | 1.24 (1.10-1.39)  1.18 (1.04-1.32)  1.11 (0.99-1.25) | 1.00  1.00  1.00 |
| Breast cancer  (in women) | 15-44  45-69  70-79  80+ | 1.25 (1.20-1.30)  1.34 (1.29-1.39)  1.25 (1.21-1.30)  1.16 (1.11-1.20) | 1.13 (1.04-1.22)  1.13 (1.04-1.22)  1.09 (1.01-1.18)  1.06 (0.98-1.15) | 1.00  1.00  1.00  1.00 |
| Colon cancer | 15-69  70-79  80+ | 1.68 (1.55-1.82)  1.48 (1.36-1.60)  1.30 (1.20-1.40) | 1.18 (1.05-1.33)  1.13 (1.01-1.27)  1.09 (0.97-1.22) | 1.00  1.00  1.00 |
| *Relative risks of ischaemic heart disease and ischaemic stroke due to diabetes are 2.19 (1.81-2.66) and 2.64 (1.78-3.92) respectively [6].  NB. Values shown are the mean and 95% confidence intervals. | | | | |

Figure III: Prevalence of physical activity in Australia (derived from data in the National Health Survey 2004-05)

The intervention impact on disease is quantified by a modified version of the PIF, in which the intervention effect changes the *relative risk* of disease in the intervention population rather than the population *prevalence* of physical activity (Equation 2).

(2)

where:

p*_i_* is the prevalence of physical activity at level *i*;

RR*_i_* is the relative risk of disease associated with physical activity at level *i*; and

RR'*_i_* is the relative risk of disease associated with physical activity after an intervention is implemented in the population.

The PIF is modified to accommodate intervention studies that measure physical activity outcomes on a variety of continuous scales (e.g. minutes per week, pedometer step counts) rather than taking broad categorical approaches (e.g. active or inactive). Each of the intervention outcome measures is translated into a change in energy expenditure (Text S1), from which a change in relative risk of disease is derived.

The change in relative risk is derived by assuming relative risks decrease linearly with increasing energy expenditure, based on relative risk (Table I) and mean energy expenditure (Table II) values for the inactive and insufficiently active categories of physical activity. Above a *sufficient* level of physical activity it is assumed that there is no excess risk. Definition of *sufficient* physical activity is based on the Australian Institute of Health and Welfare definition of 2 ½ hours per week of moderate activity that is accumulated over at least five separate sessions [7]. *Moderate* activity can be defined as physical activity that produces an energy expenditure between three and six times the resting metabolic rate of 1 MET = 1kcal/kg/hr [8]; we apply a value of 5 METs, Australia’s National Health Survey definition of moderate activity [9]. Combining these definitions leads to an energy expenditure of 750 MET-minutes per week for *sufficient* physical activity. For the average adult Australian of 80 kg, this equates to an energy expenditure of 1000 kilocalories per week, a level estimated to be sufficient for avoiding premature mortality [10,11].

Table II: Mean energy expenditure in MET-minutes per week by physical activity category (derived from data in the National Health Survey 2004-05)

|  | **Inactive** | **Insufficient** | **Sufficient** |
| --- | --- | --- | --- |
| Males | 4 | 401 | 2012 |
| Females | 5 | 393 | 1701 |

**Disease costs**

Cost offsets, due to reduced rates of physical activity-related diseases, are evaluated using disease treatment costs from the Australian Institute of Health and Welfare Disease Costs and Impacts Study 2001 (Table III). The average cost per incident case (breast cancer and colon cancer) or prevalent case (ischaemic heart disease, stroke and diabetes) is derived for each modelled disease, based on rates of disease in 2001 [2], and adjusted to the year 2003 using the Australian Health Price Index [12].

Health care costs for all other diseases and injuries, which will accrue in added years of life, are also derived from the Disease Costs and Impacts Study data [13]. These costs are derived as a cost per person, by age and sex, excluding costs of the physical activity-related diseases explicitly modelled.

Table III: Cost per prevalent or incident case of disease.

| **Age** | **Ischaemic heart disease*** | **Stroke*** | **Type 2 diabetes*** | **Breast cancer**** | **Colon cancer**** |
| --- | --- | --- | --- | --- | --- |
| Males |  |  |  |  |  |
| <55 | $2,962 | $2,228 | $504 | – | $17,490 |
| 55–64 | $1,988 | $4,942 | $660 | – | $17,657 |
| 65–74 | $1,664 | $9,529 | $763 | – | $18,164 |
| 75-84 | $1,512 | $12,856 | $639 | – | $18,037 |
| 85+ | $1,394 | $16,301 | $594 | – | $19,288 |
| Females |  |  |  |  |  |
| <55 | $1,832 | $1,161 | $506 | $515 | $17,136 |
| 55–64 | $1,520 | $2,090 | $759 | $509 | $16,349 |
| 65–74 | $1,595 | $5,106 | $839 | $638 | $17,238 |
| 75-84 | $1,564 | $13,137 | $745 | $739 | $17,360 |
| 85+ | $1,670 | $19,679 | $429 | $700 | $16,545 |
| * Cost per prevalent case of disease.  ** Cost per incident case of disease.  NB. Costs are in Australian dollars, adjusted to the year 2003. | | | | | |

**References**

1. Barendregt JJ, Van Oortmarssen GJ, Van Hout BA, Van Den Bosch JM, Bonneux L (1998) Coping with multiple morbidity in a life table. Mathematical Population Studies 7: 29-49.

2. Begg S, Vos T, Goss J, Barker B, Stevenson C, et al. (2007) The burden of disease and injury in Australia 2003. Canberra: Australian Institute of Health and Welfare.

3. Bull F, Armstrong T, Dixon T, Ham S, Neiman A, et al. (2004) Physical inactivity. In: Ezzati M, Lopez A, Rodgers A, Murray C, editors. Comparative Quantification of Health Risks: Global and Regional Burden of Disease Attributable to Selected Major Risk Factors. Geneva: World Health Organisation. pp. 729-881.

4. Barendregt J, Van Oortmarssen G, Vos T, Murray C (2003) A generic model for the assessment of disease epidemiology: the computational basis for DisMod II. Population Health Metrics 1: 4.

5. ABS (2005) National Health Survey - Confidential Unit Record Files, 2004-05. Canberra: Australian Bureau of Statistics.

6. Woodward M, Zhang X, Barzi F, Pan W, Ueshima H, et al. (2003) The effects of diabetes on the risks of major cardiovascular diseases and death in the Asia-Pacific region. Diabetes Care 26: 360-366.

7. AIHW (2004) Physical activity, diet and body weight: results form the 2001 National Health Survey. Canberra: Australian Institute of Health and Welfare.

8. Pate RR, Pratt M, Blair SN, Haskell WL, Macera CA, et al. (1995) Physical activity and public health - a recommendation from the Centers for Disease Control and Prevention and the American College of Sports Medicine. JAMA-Journal of the American Medical Association 273: 402-407.

9. ABS (2001) National Health Survey: Users' Guide. Canberra: Australian Bureau of Statistics.

10. Lee I-M, Skerrett PJ (2001) Physical activity and all-cause mortality: what is the dose-response relation? Medicine and Science in Sports and Exercise 33: S459-S471.

11. Oguma Y, Sesso HD, Paffenbarger RS, Jr, Lee I-M (2002) Physical activity and all cause mortality in women: a review of the evidence. British Journal of Sports Medicine 36: 162-172.

12. AIHW (2006) Health expenditure Australia. Canberra: Australian Institute of Health and Welfare.

13. AIHW (2001) Disease costs and impacts study data. Canberra: Australian Institute of Health and Welfare.
