# Supplementary material for: Cost-Effectiveness of Interventions to Promote Physical Activity: A Modelling Study
Source: PLoS Med. 2009 Jul 14;6(7):e1000110. doi: 10.1371/journal.pmed.1000110 (PMC2700960; doi:10.1371/journal.pmed.1000110)
Supplement: Text S3 — Input parameters and uncertainty. (0.15 MB DOC) [file pmed.1000110.s003.doc]

Table I: Uncertainty around costing parameters.

| **Parameter** | **Value**  **Mean (Stand dev.)** | **Distribution** | **Sources and assumptions** | **Interventions** |
| --- | --- | --- | --- | --- |
| Mass media campaign | $13.3 million ($1.33 million) | Gamma | Cost of NSW campaign [1], scaled to population. Standard error assumed to be 10% of point-estimate. | Mass media |
| Internet site | $21 million ($17 million) | Gamma | Mean annual cost of providing health information website [2-5] | Internet |
| TravelSmart | $83 ($11) per household | Gamma | Average per household cost of implementing TravelSmart in WA [6-10] | TravelSmart |
| GP prescription set-up & co-ordination | $79.66 ($7.97) per patient | Gamma | Per patient cost for Green Prescription program [11]. Standard error assumed to be 10% of point-estimate. | GP prescription |
| GP prescription exercise physiologist support | $66.49 ($6.65) per patient | Gamma | Per patient cost for Green Prescription program [11]. Standard error assumed to be 10% of point-estimate. | GP prescription |
| Exercise physiologist | $52,568 ($2,495) per annum  $26.51 ($1.26) per hour | Triangular | District Health Services Employees' Award - State, QLD Health Professional Stream Wage Rates (Level PO2 – PO3) with minimum of 4 years experience (www.health.qld.gov.au/industrial_relations/wage_rates_professional.asp) | GP referral (per annum); Pedometers (per hour) |
| Project officer | $63,323 ($1,847) per annum | Triangular | Advertised salary for mental health project officer with Australian Division of General Practice (www.adgp.com.au/ client_images  /78061.doc) | GP referral |
| Study nurse | $31.13 ($0.49) per hour (casual rate)  $25.31 ($0.40) per hour (hourly rate) | Triangular | Nurses (Queensland Public Health Sector) Award 2004 (Level 1) with average of 5 years experience (www.health.qld.gov.au/  industrial_relations/wage_rates_nursing.asp) | GP referral (casual rate);  Pedometers (hourly rate) |
| Printing | $0.50 ($0.05) per information pack and/or form | Gamma | Cost of 8 page, colour, A4 booklet from The Online Printer (www.theonlineprinter.com.  au/quote_dis_booka4.aspx). Standard error assumed to be 10% of point-estimate. | GP referral; Pedometers |
| Pedometers | $41.44 ($9.40) per pedometer | Gamma | Yamax brand pedometer price range (www.healthmg.com.au/type.idc?Ptype=  Pedometers&start=0) | Pedometers |
| Mail-out | $0.43 ($0.04) per letter  $54.00 ($5.38) annual fee | Gamma | Australia Post charges for small postage paid letter and annual fee for reply paid number. Standard error assumed to be 10% of point-estimate. | Pedometers |
| Disease cost offsets | See Table 3 in Text S2 | Uniform | Australian Institute of Health and Welfare Disease Costs and Impacts Study 2001. Minimum/maximum assumed to be ±25% of point-estimate. | All interventions |
| Exercise physiologist time in consultation | 75% (6%) | Triangular | Estimate | GP referral |
| Session times:  Screening  Initial  Follow-up | 13 mins (1.0 mins)  35 mins (2.0 mins)  20 mins (1.6 mins) | Triangular | Trial data of 10 – 15 minutes, 30 – 40 minutes and 20 minutes [12,13] | GP referral |
| Time taken by patient to complete questionnaire | 30 mins (2.5 mins) | Triangular | Estimate | GP referral |
| Time taken by nurse to screen questionnaire | 5 mins (0.4 mins) | Triangular | Estimate | GP referral |
| Time taken by GP in signing letters, etc. | 120 mins (9.8 mins) | Triangular | Estimate | GP referral |
| On-costs loading for personnel | 1.6 (0.04) | Triangular | Includes administrative assistance (e.g. with phone calls), stationery & travel | GP referral |
| Patient contribution to GP fees | 15% (2%) | Triangular | Medicare Benefits Schedule Nov 2004. Minimum/maximum assumed to be ±5% of point-estimate. | GP referral |
| NB. All costs adjusted to 2003 Australian dollars. | | | | |

Table II: Uncertainty around parameters for evaluating health effects.

| **Parameter** | **Value**  **mean (stand dev.)** | **Distribution** | **Sources and assumptions** | **Interventions** |
| --- | --- | --- | --- | --- |
| Relative risks (RRs) of disease | See Table 2 in Text S2 | Normal (ln RR) | Physical activity risks [14]  Diabetes risks [15] | All interventions |
| Before-after difference in physical activity | Intervention:  0.19 (0.16) hrs/wk  Comparator:  -0.45 (0.13) hrs/wk | Normal | NSW mass media campaign [16]. | Mass media |
| Intensity of activity in 25-60 year olds | 3.8 (0.57) METs | Log-normal | National Health Survey 2004-05 | Mass media |
| Change in activity due to intervention | 2.67 (1.12) kcal/kg/wk | Normal | Green prescription trial [17] | GP prescription |
| Change in mins per session & sessions per wk | See data in Table 1 in Text S1 (quartiles) | Cumulative* | GP referral trial [13] | GP referral |
| Change in activity due to intervention | 129 (49) MET-mins/wk | Normal | Meta-analysis of three trials [18-20] | Internet |
| Change in activity due to intervention | 57 (34) MET-mins/wk | Normal | Weighted average of TravelSmart effects for 20 locations [21-37] | TravelSmart |
| Change in activity due to intervention | 2,491 (711) steps/day | Normal | Systematic review and meta-analysis [38] | Pedometers |
| * A continuous user-defined distribution | | | | |

Table III: Uncertainty around intervention recruitment parameters.

| **Parameter** | **Value**  **mean (stand dev.)** | **Distribution** | **Sources and assumptions** | **Interventions** |
| --- | --- | --- | --- | --- |
| General practice recruitment | GP prescription:  61% (6.1%)  GP referral:  65% (6.5%) | Beta | GP prescription: Green prescription trial [17]  GP referral: Medicare Practice Incentive Program statistics | GP prescription; GP referral |
| General practice size | 5.4 (2.6) GPs per practice | Cumulative* | BEACH data in General Practice Activity in Australia 2003-4 [39] | GP prescription; GP referral |
| GP prescription recruitment and intervention completion rates | Medically excluded:  1.0% (0.1%)  Miss/decline screening:  12% (1.2%)  Decline to participate:  33% (3.3%)  Ineligible:  4.6% (0.5%)  Completion:  85% (8.5%) | Beta | Green prescription trial [17]. Standard error assumed to be 10% of point-estimate. | GP prescription |
| GP referral recruitment and intervention completion rates | Complete screening:  32% (3.2%)  Ineligible:  52% (5.2%)  Attend at 3 months:  92% (9.2%)  Attend at 6 months:  90% (9.0%)  Completion:  88% (8.8%) | Beta | GP referral trial [13]. Standard error assumed to be 10% of point-estimate. | GP referral |
| Internet participation & attrition rates | Participation:  17% (7.9%)  Attrition:  18% (5.9%) | Beta | Participation: weighted average of two trials [19,20] (rate not reported in one trial [18]).  Attrition: weighted average of three trials [18-20] | Internet |
| TravelSmart household participation rates | Contacted:  83% (15%)  Participate:  91% (4%) | Beta | Average rates from TravelSmart evaluation in 20 locations [21-37] | TravelSmart |
| Change in pedometer use due to intervention | 13% (1.3%) | Beta | Difference in rate of pedometer use between intervention and comparator communities in Rockhampton 10,000 steps study [40]. | Pedometers |
| * A continuous user-defined distribution | | | | |

References

1. NSW Health (2000) Public education campaign to promote physical activity among older people: NSW evaluation report. NSW Department of Health.

2. Department of Human Services (2002) Annual Report 2001-02. Victorian Government.

3. Department of Human Services (2003) Annual Report 2002-03. Victorian Government.

4. Department of Human Services (2004) Annual Report 2003-04. Victorian Government.

5. Department of Human Services (2005) Annual Report 2004-05. Victorian Government.

6. DPI (2003) Annual Report 2002-03. Department for Planning and Infrastructure, Government of Western Australia.

7. DPI (2004) Annual Report 2003-04. Department for Planning and Infrastructure, Government of Western Australia.

8. DPI (2005) Annual Report 2004-05. Department for Planning and Infrastructure, Government of Western Australia.

9. DPI (2006) Annual Report 2005-06. Department for Planning and Infrastructure, Government of Western Australia.

10. DPI (2007) Annual Report 2006-07. Department for Planning and Infrastructure, Government of Western Australia.

11. Elley CR, Kerse N, Arroll B, Swinburn B, Ashton T, et al. (2004) Cost effectiveness of physical activity counselling in general practice. NZMJ 117: U1216.

12. Halbert JA, Silagy CA, Finucane P, Withers RT, Hamdorf PA (1999) Recruitment of older adults for a randomized, controlled trial of exercise advice in a general practice setting. Journal of the American Geriatrics Society 47: 477-481.

13. Halbert JA, Silagy CA, Finucane PM, Withers RT, Hamdorf PA (2000) Physical activity and cardiovascular risk factors: effect of advice from an exercise specialist in Australian general practice. Medical Journal of Australia 173: 84-87.

14. Bull F, Armstrong T, Dixon T, Ham S, Neiman A, et al. (2004) Physical inactivity. In: Ezzati M, Lopez A, Rodgers A, Murray C, editors. Comparative Quantification of Health Risks: Global and Regional Burden of Disease Attributable to Selected Major Risk Factors. Geneva: World Health Organisation.

15. Woodward M, Zhang X, Barzi F, Pan W, Ueshima H, et al. (2003) The effects of diabetes on the risks of major cardiovascular diseases and death in the Asia-Pacific region. Diabetes Care 26: 360-366.

16. Bauman AE, Bellew B, Owen N, Vita P (2001) Impact of an Australian mass media campaign targeting physical activity in 1998. American Journal of Preventive Medicine 21: 41-47.

17. Elley CR, Kerse N, Arroll B, Robinson E (2003) Effectiveness of counselling patients on physical activity in general practice: cluster randomised controlled trial. BMJ 326: 793-.

18. Kosma M, Cardinal BJ, McCubbin JA (2005) Longitudinal effects of a web-based physical activity motivational program among adults with physical disabilities. Research Quarterly for Exercise and Sport 76: A116-A116.

19. Napolitano MA, Fotheringham M, Tate D, Sciamanna C, Leslie E, et al. (2003) Evaluation of an Internet-based physical activity intervention: A preliminary investigation. Annals of Behavioral Medicine 25: 92-99.

20. Plotnikoff RC, McCargar LJ, Wilson PM, Loucaides CA (2005) Efficacy of an e-mail intervention for the promotion of physical activity and nutrition behavior in the workplace context. American Journal of Health Promotion 19: 422-429.

21. Socialdata Australia (2003) Final Report: TravelSmart® Households - Marangaroo. Western Australia: Department for Planning and Infrastructure.

22. Socialdata Australia (2003) Final Report: TravelSmart® Households - Cambridge. Western Australia: Department for Planning and Infrastructure.

23. Socialdata Australia (2004) Final Report: TravelSmart® Households - City of Subiaco. Western Australia: Department for Planning and Infrastructure.

24. Socialdata Australia (2004) Final Report: TravelSmart® Households - City of Freemantle. Western Australia: Department for Planning and Infrastructure.

25. Socialdata Australia, Institute for Transport and Infrastructure Research Ltd (2004) Final Report: TravelSmart® Darebin. Victoria: Department for Infrastructure.

26. Socialdata Australia, Institute for Transport and Infrastructure Research Ltd (2004) Final Report: TravelSmart® Alamein. Victoria: Department for Infrastructure.

27. Socialdata Australia (2005) Final Report: TravelSmart® Households - City of Belmont. Western Australia: Department for Planning and Infrastructure.

28. Socialdata Australia (2005) Final Report: TravelSmart® Households - Town of Vincent. Western Australia: Department for Planning and Infrastructure.

29. Socialdata Australia (2005) Final Report: TravelSmart® Households - City of Armadale. Western Australia: Department for Planning and Infrastructure.

30. Socialdata Australia (2007) TravelSmart® Household Final Evaluation Report: City of Wanneroo - Clarkson and Surrounds. Western Australia: Department for Planning and Infrastructure.

31. Sustrans (2004) TravelSmart Bristol (Bishopston). UK: TravelSmart.

32. Sustrans (2004) TravelSmart Gloucester (Quedgeley). UK: TravelSmart.

33. Sustrans (2004) TravelSmart Sheffield (Hillsborough/Middlewood) 2003-04. UK: TravelSmart.

34. Sustrans (2004) Greater Nottingham TravelSmart (Lady Bay and the Meadows). UK: TravelSmart.

35. Sustrans (2004) TravelSmart Northumberland (Cramlington). UK: TravelSmart.

36. Socialdata America (2004) IndiMark® and Behavior Change Results: For the Cities of Salem-Keizer, Eugene, and Bend, Oregon. Oregon Department of Transportation.

37. Tapestry (2003) Viernheim Household Transport. Germany.

38. Bravata DM, Smith-Spangler C, Sundaram V, Gienger AL, Lin N, et al. (2007) Using pedometers to increase physical activity and improve health - A systematic review. Jama-Journal of the American Medical Association 298: 2296-2304.

39. Britt H, Miller G, Knox S, Charles J, Valenti L, et al. (2004) General practice activity in Australia 2003–04. Canberra: Australian Institute of Health and Welfare.

40. Eakin EG, Mummery K, Reeves MM, Lawler SP, Schofield G, et al. (2007) Correlates of pedometer use: Results from a community-based physical activity intervention trial (10,000 Steps Rockhampton). International Journal of Behavioral Nutrition and Physical Activity 4.
